# Lethal and sublethal synergistic effects of a new systemic pesticide, flupyradifurone (Sivanto^®^), on honeybees

**DOI:** 10.1098/rspb.2019.0433

**Published:** 2019-04-10

**Authors:** S. Tosi, J. C. Nieh

**Affiliations:** 1 Division of Biological Sciences, Section of Ecology, Behavior, and Evolution, University of California, San Diego, CA, USA; 2 Epidemiology Unit, European Union Reference Laboratory (EURL) for Honeybee Health, University Paris Est, ANSES (French Agency for Food, Environmental and Occupational Health and Safety) Animal Health Laboratory, Maisons-Alfort, France

**Keywords:** seasonality, bee age, chemical mixture, EBI fungicide, nAChR, behaviour

## Abstract

The honeybee (*Apis mellifera* L.) is an important pollinator and a model for pesticide effects on insect pollinators. The effects of agricultural pesticides on honeybee health have therefore raised concern. Bees can be exposed to multiple pesticides that may interact synergistically, amplifying their side effects. Attention has focused on neonicotinoid pesticides, but flupyradifurone (FPF) is a novel butenolide insecticide that is also systemic and a nicotinic acetylcholine receptor (nAChR) agonist. We therefore tested the lethal and sublethal toxic effects of FPF over different seasons and worker types, and the interaction of FPF with a common SBI fungicide, propiconazole. We provide the first demonstration of adverse synergistic effects on bee survival and behaviour (poor coordination, hyperactivity, apathy) even at FPF field-realistic doses (worst-case scenarios). Pesticide effects were significantly influenced by worker type and season. Foragers were consistently more susceptible to the pesticides (4-fold greater effect) than in-hive bees, and both worker types were more strongly affected by FPF in summer as compared with spring. Because risk assessment (RA) requires relatively limited tests that only marginally address bee behaviour and do not consider the influence of bee age and season, our results raise concerns about the safety of approved pesticides, including FPF. We suggest that pesticide RA also test for common chemical mixture synergies on behaviour and survival.

## 1. Introduction

Pollinators provide ecosystem services that are crucial for crop production and wild plant biodiversity [1]. The honeybee is a major pollinator [2] whose global decline in health raises concerns about ecological impacts, including food security and human welfare [3]. Multiple studies have focused on honeybees because their general biochemistry and neurophysiology are better known than other pollinators and since bees can be used to model pesticide harm to other insect pollinators [4]. Pesticides are among the most important stressors affecting bee health [5] and pose heightened risks when bees are exposed to multiple pesticides for extended periods [6]. Attention has focused on the neonicotinoid pesticides [7], but their use has been progressively restricted because of their adverse effects on bees [8], and growing pesticide resistance [9]. New pesticides, such as flupyradifurone (FPF, Sivanto^®^, Bayer CropScience AG [10]), have therefore entered the market [9].

FPF is a newly developed systemic insecticide [11] that shares multiple similarities with the neonicotinoids. Its chemical structure partially overlaps with neonicotinoids, but FPF is a butenolide insecticide because of its different pharmacophore [12]. Nonetheless, they share the same target site (agonists of insects nAChRs, Insecticide Resistance Action Committee (IRAC) group 4), are both systemic [12] and control a wide variety of pests [12] on diverse crops through multiple application methods [10,12]. FPF metabolites include 6-chloronicotinic acid, which is also a metabolic by-product of most neonicotinoids [9], and can cause adverse oxidative stress in organisms such as freshwater amphipods and algae [13].

Because FPF is relatively new, fewer pest species are resistant to it as compared with the neonicotinoids [11,12,14]. FPF is also thought to have a favourable ecotoxicological safety profile [12] and is defined as relatively ‘bee safe’ [15]. Consequently, while neonicotinoids can only be used to treat crops in the absence of bee foraging, FPF can be used on flowering crops when bees are actively foraging.

Relatively few studies have investigated the impacts of FPF on bees. Hesselbach & Scheiner [16] showed that acute exposure to a high, non-field-realistic FPF dose (1.2 µg bee^−1^) impaired bee taste and cognition. Tan *et al*. [17] demonstrated that chronic exposure to FPF impaired olfactory learning in larval (0.033 µg larvae d^−1^) and adult (0.066 µg adult bee d^−1^) Asian honeybees (*Apis cerana*) at field-realistic doses. Campbell *et al*. [18] tested the effects of FPF in a USA field study and observed no significant side effects on bee colony strength. This latter study, however, shows the difficulty of performing ecotoxicological field trials [19]: bee-collected nectar and pollen sampled from control fields were also contaminated with FPF.

Synergistic effects occur when combined exposure to two factors results in an effect that is significantly greater than the sum of individual effects. Such synergistic effects can occur between pesticide and poor nutrition, reducing bee survival, food consumption and energy levels [20]. Synergy can also arise between a pesticide and disease [21] or from exposure to multiple pesticides [22]. Chemical mixtures can have sublethal effects that do not immediately reduce survival [23,24]. For example, pesticide mixtures can synergistically alter behaviour such as mobility in aquatic organisms [25,26]. The combination of diseases (fungi) and pesticide (imidacloprid) can synergistically alter beetle movement [27]. However, there is, to date, no evidence that pesticides have significant synergistic effects on pollinator behaviour.

Neonicotinoids and SBI (sterol biosynthesis inhibitor) fungicides have synergistic effects on bees because SBI fungicides can inhibit detoxification [28]. FPF and the fungicide, tebuconazole, decreased FPF LD_50_ of in-hive bees by 6-fold [15]. The SBI fungicide propiconazole (PRO; chemical group: triazole; MoA code: G1, DeMethylation Inhibitors (DMI), SBI class 1; Fungicide Resistance Action Committee (FRAC) code: 3) is one of the most commonly used fungicides and is found in the environment and in bee food [29–32]. Both FPF and PRO are used on the same common crops [33]. As FPF is a relatively new pesticide, no monitoring studies have yet tested its co-occurrence as an environmental contaminant with other pesticides. However, FPF is approved for many of the same crops as the neonicotinoids [33], and neonicotinoids co-occur with SBI fungicides in bee pollen [6]. We therefore investigated the potential synergistic effects of two systemic pesticides, FPF and PRO.

We also examined the effects of seasonality. Although summer bees are typically more sensitive to pesticides than winter bees [34–37], some studies have reported different results [35,38]. Baines *et al.* [38] showed that early spring bees (March) were more susceptible to pesticides than summer bees. Decourtye *et al*. [35] found that exposure to a neonicotinoid pesticide (imidacloprid) reduces survival of winter bees, but reduced learning performances of summer bees. Seasons influence the floral and food resources available, thereby altering pesticide resistance [20], toxicokinetics and bee immunity [39]. Pesticide effects are also temperature dependent and alter thermoregulation [40], and season could alter bee detoxification abilities [39].

The effects of pesticides can be influenced by the age and body weight of an organism [39,41,42]. In-hive bees are typically heavier and more resistant to pesticides than foragers [43]. Bee responses to toxins also change as they age [44–48], and older bees may be more sensitive to pesticides [34]. However, to date, no studies have examined how the toxicity of FPF varies across worker type and season. We thus tested the effects of FPF over seasons and between worker types, assessing its interactive effects with a common SBI fungicide, PRO, on bee behaviour and survival.

## 2. Material and methods

We used six healthy honeybee colonies (*Apis mellifera ligustica* Spinola, 1806, located at the UCSD Biology Field Station apiary, La Jolla, USA), studied forager and in-hive honeybees, and followed standard collection and rearing methodologies [49]. To test the effect of season, we collected bees at two different colony developmental stages: early spring (February–March 2016) and summer (July 2016). We tested the synergistic and individual effects of FPF exposing bees to five acute oral doses of FPF or FPF + PRO. Based on current guidelines [50,51], we tested FPF doses (375 and 750 ng bee^−1^) considered field-realistic, since bees can ingest higher FPF doses while foraging (see electronic supplementary material for the worst-case scenario estimations).

Following previous studies [22,28,52], we used a relatively high PRO dose that nonetheless, on its own, has no impact on bee survival (7000 ng bee^−1^ [22,52]). PRO is one of the most commonly used fungicides that contaminates bees and the environment [31,32]. Bees can be simultaneously exposed to FPF and PRO (or another SBI fungicide with similar mode of action) because they are used on the same crops and ornamentals, including fruits (e.g. citrus), oilseeds (e.g. soya bean, peanuts), cereals (e.g. corn, sorghum) [10,12,15,53–55], although guidelines state that flupyradifurone should not be directly tank-mixed with azole fungicides when applied to flowering crops [10]. These pesticides can be used multiple times over a year in the same crop (and over different seasons) and applied in multiple ways (i.e. aerial, chemigation or ground application). In addition, bees can also be exposed to pesticides that drift from different crops (i.e. buffer zones) or are stored in the same hive [56,57]. FPF and PRO are easily taken up by plants and thus contaminated soil and water may lead to unintended absorption. This can result in prolonged, multi-year contamination [56,58,59]. Bees can therefore be exposed to pesticide combinations that are contraindicated in tank mixes [6].

We tested a control dose (0 ng bee^−1^), a total of six doses of FPF (375, 750, 1500, 3000, 6000, 12 000 ng bee^−1^, respectively corresponding to 37.5, 75, 150, 300, 600, 1200 ppm), and five doses of the positive control dimethoate (DIM; 50, 100, 200, 400, 800 ng bee^−1^, respectively corresponding to 5, 10, 20, 40, 80 ppm). In the combined FPF + PRO treatment, each FPF dose was tested in combination with a single sublethal dose of PRO (7000 ng bee^−1^, corresponding to 700 ppm). We used technical grades of all active ingredients. The test solutions (sucrose 50% w/w, 100 µl cage^−1^, 10 µl bee^−1^,) were provided inside each cage using an Eppendorf cap [60], contained acetone as a solvent (0.7%) and were completely consumed 60 min after oral administration [60].

We measured the effects of treatment on bee survival (1–48 h) and the frequency of bees exhibiting abnormal behaviours (1–4 h, see below).

Detailed methods are reported in the electronic supplementary material.

### (a) Abnormal behaviours: synergistic and individual effects

We measured the percentage of bees exhibiting abnormal behaviours (i.e. the number of abnormally behaving bees per cage) across time (1, 2 and 4 h after treatment) depending on pesticide dose, season and worker type. We quantified the following behaviours: motion coordination deficits, hyperactivity, apathy, curved-down abdomen or moribund (electronic supplementary material, table S1) [4,15,51,60–62]. These abnormal behaviour categories are based on official ecotoxicological guidelines [60,63]. The unit of replication was the cage, and we observed each bee for 6 s (a maximum of 60 s for a cage with 10 bees). To improve the standardization and repeatability of our behavioural assessments, we refined the accuracy of the definitions of the behaviours of the official ecotoxicological guidelines [60,63] through videos (electronic supplementary material) and descriptions (electronic supplementary material, tables S1 and S2). We measured abnormal behaviours up to 4 h because high mortality at later time points severely reduced sample sizes in certain treatments and because behavioural abnormalities primarily occurred less than 4 h after treatment. The experimenters were blind to the treatments and were trained using standard descriptions (electronic supplementary material, tables S1 and S2) and videos (electronic supplementary material) of the behaviours. Before being allowed to score behaviours, experimenters needed to score standard videos with greater than 95% consistency.

### (b) Statistical analysis

To test for synergy, we determined if the difference between the expected and the observed effects (either mortality or presence of abnormal behaviour) of the combined treatment could arise by chance alone or was larger than the simple additive effect of both pesticides.

We used the concentration addition (CA) reference model to define biologically significant synergy of chemical mixtures [64]. Based on each worker type LD_50_, we calculated the model deviation ratio (MDR) to determine if the FPF + PRO interaction caused synergistic (MDR > 2), additive (0.5 ≤ MDR ≤ 2), or antagonistic (MDR < 0.5) effects [25]. To estimate the MDR, we calculated the toxic unit (TU) of each individual pesticide (FPF, PRO) and of the binary chemical mixture (FPF + PRO) [51].

We calculated the risk ratio (RR) and the risk difference (RD) to quantitatively express both relative (RR) and absolute (RD) size of the interactive effect of the chemical mixture on bee survival (frequency of dead bees; electronic supplementary material, table S3) and behaviour (frequency of abnormally behaving bees; electronic supplementary material, table S4) [65,66]. The RR was determined by dividing the observed effects by the expected effects and therefore cannot be calculated when the expected effect is 0 [65,66]. The RD is the difference between the ratio of observed and expected effects.

## 3. Results

### (a) Pesticides synergistically increased mortality

**Figure 1.**
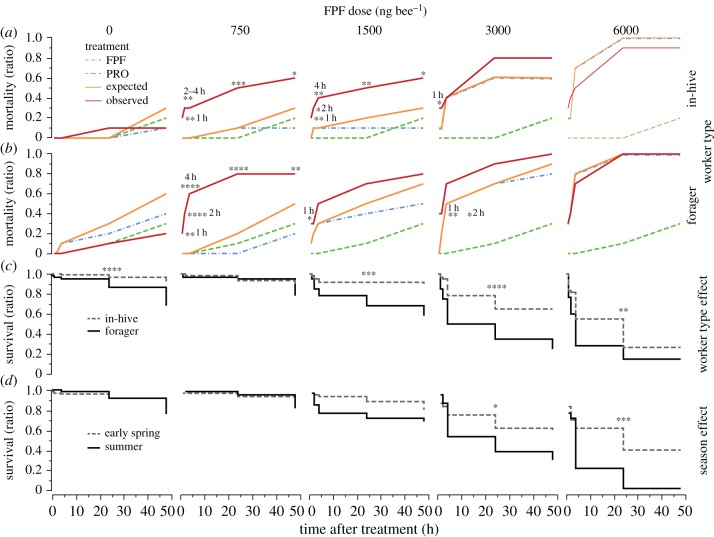
Lethal effects of FPF vary based on (*a*,*b*) combination with another pesticide (PRO), (*c*) bee worker type and (*d*) season. In (*a*,*b*), lethal synergistic effects of FPF + PRO on bee survival across time and worker type ((*a*) in-hive bees; (*b*) foragers). We tested the individual effects of FPF (blue dashed lines) and PRO (green dashed lines) and compared their expected (orange full lines) and observed (red full lines) combined effects, on bee mortality. In (*c*,*d*), we show the influence of (*c*) worker type and (*d*) season on bee sensitivity to FPF doses, assessed as survival across time. Asterisks indicate significant (*a*,*b*) synergistic (significant difference between mortality of expected and observed combined treatment; binomial proportion tests, Holm corrected; *n* = 390; electronic supplementary material, table S3) or (*c*,*d*) individual (Kaplan–Meier^DS^; *n* = 1440; electronic supplementary material, table S6) effects of FPF at specific time assessments (*p = 0.05, **p = 0.01, ***p = 0.001, ****p = 0.0001).

The combination of FPF and PRO (FPF + PRO) synergistically increased mortality of both in-hive and forager bees (binomial proportion test, Holm correction; electronic supplementary material, table S3; figure 1*a,b*). The synergistic effect of FPF + PRO significantly reduced in-hive bee survival at 750 ng bee^−1^ (1–48 h after exposure, RR_Max_ = 9, RD_Max_ = 44; electronic supplementary material, table S3), 1500 ng bee^−1^ (1–48 h after exposure, RR_Max_ = 5, RD_Max_ = 37) and 3000 ng bee^−1^ of FPF (1 h after exposure, RR_1 h_ = 5, RD_1 h_ = 23). The synergistic effect of FPF + PRO significantly reduced forager survival at 750 ng bee^−1^ (1–48 h after exposure, RR_Max_ = 5, RD_Max_ = 64; electronic supplementary material, table S3), 1500 ng bee^−1^ (1 h after exposure, RR_Max_ = 5, RD_Max_ = 27) and 3000 ng bee^−1^ of FPF (1–2 h after exposure, RR_Max_ = 11, RD_Max_ = 33).

These synergistic effects were weaker at higher doses (electronic supplementary material, tables S3 and S4): FPF alone caused higher mortality at increasing doses, approaching the upper threshold of 100%, and thus reducing the difference between combined and individual treatments. Because synergy is better captured when the mortality of individual treatments is low, the synergistic effect was longer lasting for in-hive bees (i.e. 1–48 h at 1500 ng bee^−1^), than for foragers (i.e. 1 h at 1500 ng bee^−1^).

The LD_50_ of FPF + PRO (TU_FPF,summer in-hive_ = 0.25; TU_FPF,summer foragers_ = 0.19; TU_PRO_ = 0.07, TU_FPF+PRO,summer in-hive_ = 0.32, TU_FPF+PRO, summer foragers_ = 0.26) was also significantly lower than that of either compound alone for both in-hive (4-fold toxicity increase, MDR = 3.1) and foragers (5-fold toxicity increase, MDR = 3.9). Because the MDRs are higher than 2, the FPF + PRO interaction was synergistic for both worker types [64]. PRO alone did not cause any significant effect on survival (*χ*^2^ = 0.6376, d.f. = 1, *p* > 0.42).

The lower field-realistic FPF dose (375 ng FPF bee 1 h^−1^) caused a 73% mortality in bees when combined with PRO. Thus, the FPF + PRO LD_50_ was lower than 375 ng FPF bee^−1^.

There was a significant effect of FPF dose on bee survival (Fit proportional hazards, *p* < 0.0001; electronic supplementary material, table S5). Pesticide-free bees survived significantly longer than bees exposed to 1500 ng bee^−1^ (Kaplan–Meier^DS^, *p* < 0.049; electronic supplementary material, table S6), 3000 ng bee^−1^ (*p* < 0.0001), 6000 ng bee^−1^ (*p* < 0.0001), 12 000 ng bee^−1^ (*p* < 0.0001) of FPF.

**Figure 2.**
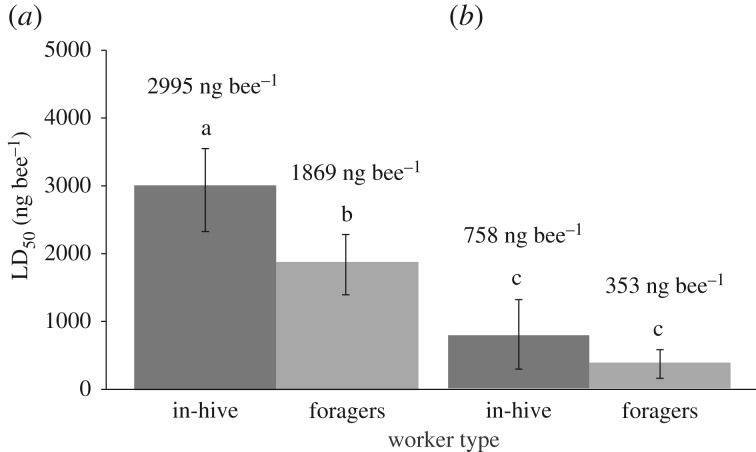
The LD_50_ (48 h) of bees exposed to FPF (*a*) and FPF + PRO (*b*) across worker types (in-hive bees versus foragers) in summer. Above each bar, we show the LD_50_ values. Different letters indicate significant differences. We show the 24 h LD_50_ of foragers (light grey bars), because high summer forager mortality at 48 h prevented the accurate estimation of their 48 h LD_50_ (standard LD_50_ estimation time, dark grey bars). Error bars represent 95% confidence intervals (*n*_overall_ = 1080). The LD_50_ of FPF across season and worker type is reported in electronic supplementary material, figure S1.

The FPF LD_50_ of our study (2995 ng bee^−1^) is 2.5 times higher than the value reported by the US EPA (1200 ng bee^−1^) [15] when we compare toxicity on standard individuals (in-hive summer bees [60], figure 2). However, we had a slightly different protocol than the US EPA [15]. We used 1 h exposure to 10 µl, not 6 h exposure *ad libitum*. Our positive control (DIM, reference toxin) met validity criteria because the 24 h LD_50_ of DIM was within the standard limits (0.10–0.35 µg bee^−1^) defined by official international guidelines [60]. There was no significant effect of the colony on bee survival (*p* > 0.05; electronic supplementary material, table S5).

### (b) Pesticides synergistically increased abnormal behaviours

**Figure 3.**
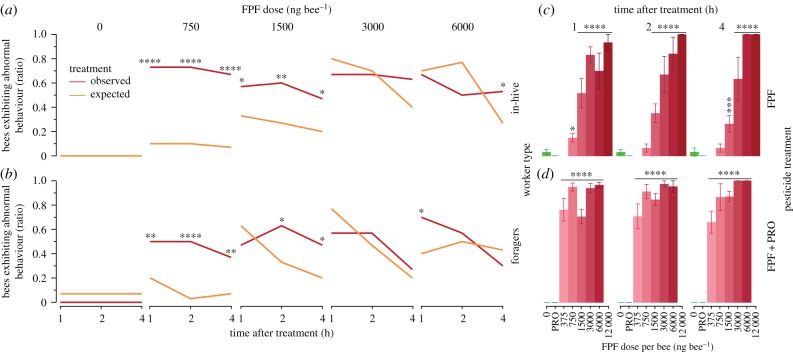
Sublethal (frequency of bees exhibiting abnormal behaviour) effects of FPF in combination with another pesticide (PRO). On the left (*a*,*b*), the synergistic effects of FPF and PRO across time (1–4 h after treatment) and worker type (in-hive bees: (*a*); foragers: (*b*)). We tested the individual effects of FPF and PRO and compared their expected (orange) and observed (red) combined effects (*a*,*b*, binomial proportion tests, Holm corrected; *n* = 390, electronic supplementary material, table S4). Because PRO alone did not alter bee behaviour alone, we only show the expected and observed results (*a,b*). On the right (*c*,*d*), sublethal effects of (*c*) FPF (750–12 000 ng FPF bee^−1^) and (*d*) FPF + PRO (375–6000 ng FPF bee^−1^, 7000 ng PRO bee^−1^) as compared with each respective control (mixed model_REML_, contrast test^DS^, Dunn–Sidak corrected; *n* = 342; electronic supplementary material, tables S7 and S8). We show the effects observed in summer, pooled by worker type in (*c*,*d*). Asterisks indicate significant effects at specific time assessments (*p = 0.05, **p = 0.01, ****p = 0.0001).

FPF + PRO synergistically increased abnormal behaviours of both in-hive and forager bees (binomial proportion test, Holm correction; electronic supplementary material, table S4; figure 3). The synergistic effect of FPF + PRO significantly increased in-hive bee abnormal behaviours at the lower FPF doses of 750 ng bee^−1^ (1–4 h after exposure, RR_Max_ = 10, RD_Max_ = 63; electronic supplementary material, table S4), 1500 ng bee^−1^ (1–4 h after exposure, RR_Max_ = 2, RD_Max_ = 33) and 6000 ng bee^−1^ of FPF (4 h after exposure, RR_4 h_ = 2, RD_4 h_ = 27). The synergistic effect of FPF + PRO significantly increased forager abnormal behaviours at 750 ng bee^−1^ (1–4 h after exposure, RR_Max_ = 15, RD_Max_ = 47; electronic supplementary material, table S4), 1500 ng bee^−1^ (2–4 h after exposure, RR_Max_ = 2, RD_Max_ = 30) and 6000 ng bee^−1^ of FPF (1 h after exposure, RR_1 h_ = 2, RD_1 h_ = 23). PRO alone did not cause any significant abnormal behaviour. As with survival (see above), the synergistic effects on abnormal behaviours were more evident at lower doses.

The lower field-realistic dose of FPF (375 ng FPF bee 1 h^−1^) significantly increased the number of bees exhibiting abnormal behaviours (RR_Max_ = 36, RD_Max_ = 80, *p* < 0.0001) when combined with PRO (electronic supplementary material, table S7; figure 3). These adverse behavioural effects started rapidly and remained consistent after treatment (RR Range_1–4 h_ = 34–36, RD_1–4 h_ = 74–80, *p* < 0.0001; table 1; electronic supplementary material, table S7).

**Table 1.** The frequency of bees exhibiting abnormal behaviours after exposure to the lower field-realistic FPF dose tested (375 ng FPF bee^−1^), combined with PRO. We report the effect size as the risk ratio (RR, observed/expected) and the risk difference (RD, observed–expected), which compared the effects of FPF + PRO (375 ng FPF bee^−1^ and 7000 ng PRO bee^−1^) versus either control (0 ng bee^−1^) or the lower dose of FPF tested alone (750 ng FPF alone per bee). Combined exposure to FPF + PRO (375 ng FPF bee^−1^) resulted in a higher frequency of bees exhibiting abnormal behaviours, even when compared with higher doses of FPF administered alone (750 ng bee^−1^; RR_range_ = 7–34; RD_range_ = 43–75). Because of the low effect of FPF alone at 375 ng FPF bee^−1^, we did not test the effects of this dose alone. We report ‘n.a.’ because the expected mortality was 0, and RR calculation is not possible when the denominator is 0.

				synergistic increase			
				versus control		versus FPF alone	
treatment	worker type	time after treatment (h)	bees exhibiting abnormal behaviour (%)	RR	RD	RR	RD
FPF + PRO (375 ng FPF bee^−1^)	in-hive	1	76	n.a.	76	34	66
		2	67	n.a.	67	30	56
		4	50	n.a.	50	22	43
	foragers	1	76	34	74	8	56
		2	74	33	72	7	71
		4	82	36	80	12	75

There was a significant effect of dose 1, 2 and 4 h after treatment with FPF (Mixed Model_REML_, *p* < 0.0001; electronic supplementary material, table S8; figure 3), DIM (*p* < 0.0001) and FPF + PRO (*p* < 0.0001). After treatment, bees showed coordination problems fairly consistent across time. Specifically, they mostly showed hyperactivity and curved-down abdomen in the shorter term after treatment (1 h) and apathy later on (4 h).

### (c) Pesticides were more toxic to foragers than in-hive bees

There was a significant effect of worker type on the survival of bees exposed to FPF and DIM (fit proportional hazards, *p* < 0.0001; electronic supplementary material, table S5). Foragers are older than in-hive bees, and thus it is not surprising that in-hive control bees lived longer than control foragers (Kaplan–Meier^DS^, *p* = 0.0001; electronic supplementary material, table S6; figure 1*c*). As the FPF dose increased, the survival of both bee castes decreased and, at each dose, the difference between the survival of each caste tended to increase (interaction FPF dose × worker type, *p* = 0.001; electronic supplementary material, table S5; figure 1*c*,*d*). FPF was significantly more toxic to foragers (compared with in-hive bees) at almost all doses tested: 1500 ng bee^−1^ (4-fold increase at 48 h, Kaplan–Meier^DS^, *p* = 0.0002; electronic supplementary material, table S6), 3000 ng bee^−1^ (2-fold, *p* < 0.0001), 6000 ng bee^−1^ (1.2-fold, *p* = 0.006) of FPF. At 12 000 ng bee^−1^ of FPF, there was no significant effect (*p* > 0.26) of worker type on survival, because the mortality of in-hive and forager bees was very high.

The LD_50_ assessments confirmed that FPF and DIM toxicity was influenced by worker type (figure 2; electronic supplementary material, figure S2). Foragers were significantly more susceptible to pesticides in both early spring (FPF: 2-fold toxicity increase; DIM: 4-fold) and summer (FPF: 2-fold; DIM: NS), as compared with in-hive bees. High forager mortality in summer prevented the estimation of the 48 h LD_50_ of summer foragers. Foragers were more affected by the synergistic effects caused by FPF + PRO (5-fold increased mortality), as compared with in-hive bees (4-fold).

There was a significant effect of worker type on bee abnormal behaviours 1 h after treatment with FPF (mixed model_REML_, *p* = 0.046; electronic supplementary material, table S3), and 1 and 2 h after treatment with DIM (*p* < 0.005). There was no significant effect of worker type at any other time point (*p* > 0.57). There was no significant effect of worker type on abnormal behaviours after exposure to FPF + PRO (*p* > 0.05).

There was a significant effect of the interaction dose × worker type after treatment with FPF (1–2 h: *p* < 0.049), DIM (1–4 h: *p* < 0.025) and FPF + PRO (4 h: *p* = 0.003; electronic supplementary material, table S3; figure 3) on bee abnormal behaviours. Foragers were more susceptible to FPF (1500 ng bee^−1^: *p* < 0.0001; electronic supplementary material, table S9) and FPF + PRO (375 ng bee^−1^: *p* < 0.006; electronic supplementary material, table S9) as compared with in-hive bees.

### (d) FPF was more toxic in summer

There was a significant effect of season on the survival of bees exposed to FPF (fit proportional hazards, *p* < 0.0001; electronic supplementary material, table S5). There was also a significant effect of the interaction FPF dose × season (*p* = 0.011). FPF was significantly more toxic in summer, as compared with early spring, at 3000 ng bee^−1^ (Kaplan–Meier^DS^, *p* = 0.013; electronic supplementary material, table S6; figure 1*d*) and 6000 ng bee^−1^ (*p* = 0.0003). At 12 000 ng of FPF, there was no significant effect of season on survival, because bee mortality was too high in both seasons (*p* > 0.78).

The LD_50_ assessments confirmed that FPF toxicity was influenced by season (electronic supplementary material, figure S1). FPF was significantly more toxic in summer, both for in-hive and forager bees (2-fold toxicity increase), as compared with early spring.

There was a significant effect of season on bee abnormal behaviours 1 and 2 h after treatment with FPF (mixed model_REML_, *p* < 0.008; electronic supplementary material, table S8). There was no significant effect of season at 4 h after treatment of FPF (*p* > 0.51). There was a significant effect of the interaction dose × season on bee abnormal behaviours after treatment with FPF (1 h: *p* = 0.0004; electronic supplementary material, table S8; figure S4). FPF significantly increased bee abnormal behaviours in summer, as compared with early spring, at 750 ng bee^−1^ (contrast test^DS^, *p* < 0.0001; electronic supplementary material, table S9; figure S4), 1500 ng bee^−1^ (*p* = 0.012) and 3000 ng bee^−1^ (*p* = 0.001).

The electronic supplementary material contains additional results, including bee weight and DIM toxicity.

## 4. Discussion

We provide the first demonstration that the combination of two pesticides can synergistically increase the frequency of pollinators with abnormal behaviours (figure 3). We also provide the first evidence of adverse synergistic lethal (MDR_Max_ = 4; RR_Max_ = 11; RD_Max_ = 64) and sublethal (RR_Max_ = 15; RD_Max_ = 63) effects caused by FPF and an SBI fungicide, PRO (electronic supplementary material, tables S3 and S4; figures 1 and 3). All FPF doses tested significantly impaired bee behaviour as compared with the control treatment (electronic supplementary material, tables S7 and S8; figure 3). FPF can thus impair bee survival and behaviour at field-realistic (worst-case) doses when combined with an SBI fungicide. In addition, the toxic effect of FPF and FPF + PRO on bee survival and behaviour was significantly influenced by worker type and season. Foragers were consistently more susceptible to these pesticides (up to 4-fold; figures 1 and 3; electronic supplementary material, figures S2–S4). This result is troubling because the official guidelines for pesticide risk assessment (RA) only test in-hive bees, thereby underestimating the risk that pesticides pose for foragers. This is also concerning given that foragers are particularly at risk of pesticide exposure since they forage in the field. The lower weight of foragers (−11%), as compared with in-hive bees (electronic supplementary material, table S10), is a possible reason for their increased susceptibility to pesticides.

Our results confirm that abnormal behaviours usually appear shortly (1 h) after exposure to pesticides [40,67,68]. However, official RA guidelines do not require a thorough assessment of abnormal behaviours, and then only 4 h after treatment, when many effects may have declined. Short-term (1–2 h) behavioural alterations can be detrimental for bee health, especially for bees carrying out risky tasks (i.e. foraging) within this time window. We demonstrated that adverse sublethal effects are more frequent in foragers (as compared with in-hive bees) and could impact their foraging efficiency, as well as their survival, if these abnormal behaviours occur while bees are foraging in the field. Future behavioural assessments conducted using standard assays such as locomotion arenas could better establish potentially harmful effects of pesticides on beneficial insects [67,69–71].

Our results show that FPF and the neonicotinoids, both of which target insect nicotinic acetylcholine receptors [9], have relatively similar effects on bee health, sharing side-effects on bee survival and behaviour [67]. The SBI fungicide, PRO, similarly amplifies both FPF (4-fold, MDR_summer in-hive_ = 3.1, this study) and neonicotinoid (3-fold [22]) toxicity in bees. With respect to survival, FPF toxicity (based on LD_50_) is over 559 times lower than the toxicity of the *N*-nitroguanidine neonicotinoids (clothianidin, imidacloprid, thiamethoxam), but more than five times higher than the *N*-cyanoamidine neonicotinoids (acetamiprid, thiacloprid) [33]. To assess the risk for bees, these differences need to be considered based on actual exposure, which depends on application methods (e.g. frequency of treatments and application rate) and active ingredient properties (e.g. toxicity).

The FPF + PRO synergistic effects on bee survival and behaviour are more evident at lower doses, where the effect of FPF is less detrimental. Our assessment suggests that FPF, like the neonicotinoids [40], may lead to a favourable biological response at low exposure levels (i.e. hormesis [72]). In foragers, the low dose of FPF (750 ng bee^−1^) resulted in reduced mortality than the control treatment (0 ng bee^−1^; figure 1*c*,*d*). Similarly, our observed synergistic behavioural alterations (figure 3) occurred at low (750–1500 ng bee^−1^) and high (6000 ng bee^−1^) doses, but not at intermediate ones (3000 ng bee^−1^). These non-monotonic effects of pesticides (i.e. hormesis) should be further investigated.

Restrictions on neonicotinoids have been increasing after decades of research demonstrating their adverse effects on beneficial pollinators and persistent environmental contamination [20,58]. New systemic insecticides such as FPF and sulfoxaflor, examples of the novel butenolide and sulfoxamine chemical classes, are the likely successors of the neonicotinoids [73]. While sulfoxaflor has adverse effects on bumblebees [74], FPF is considered ‘bee safe’ and can be used on flowering crops with actively foraging bees [12,15]. Our study raises concerns about the ecotoxicological profile of FPF and the safety of FPF for bees.

Pesticide toxicity is amplified by multiple interacting stressors [5,20] and thus a holistic approach that tests more of the different conditions that bees naturally experience is beneficial [39,75]. Although sensitivity to pesticides is influenced by season, bee type and concomitant pesticide exposure [44,76], current RA schemes implement only limited tests, requiring only the evaluation of single pesticide effects on in-hive summer bees [77]. Such restricted testing could underestimate or overestimate pesticide effects [38,77] because different worker types (i.e. in-hive bees versus foragers) can be exposed to multiple pesticides over different seasons [6]. FPF (Sivanto) labelling restricts using tank-mixtures with azole fungicides [10]. However, bees can still be exposed to FPF and PRO simultaneously, as discussed above and in the electronic supplementary material.

Future RA should consider sublethal and behavioural effects [78,79]. Studies are necessary to confirm and link laboratory results to field tests, so that specific protection goals, such as avoiding unacceptable decreases in colony population or increases in forager mortality, can be assessed [78]. Monitoring the behaviour of multiple honeybees in a whole colony, though feasible in observation colonies [80], introduces complications [19] and costs that would be likely to constrain general adoption in RA. Testing bees in the laboratory also has the benefit of greater control, ease of testing and thus replication with more colony sources.

Because RA with honeybees is often used as an indicator of potential harm to other bees [50], our results raise broader concerns. However, there can be significant differences, greater and lesser, between the individual sensitivities of different bee species, highlighting the need for future research.

Although risk assessors are beginning to address synergistic effects on survival [79,81] and sublethal effects such as homing behaviour and hypopharyngeal development [51], many important synergistic and behavioural effects that can affect colony fitness are not explored. We propose that RA include more thorough assessments of behaviours and synergies. Behavioural testing should be implemented 1 or 2 h after exposure, since short-term behavioural effects can realistically impair bee survival when ingesting pesticides while foraging [82]. RA could address potential synergies on behaviour and survival by testing limited chemical mixtures of pesticides that have a higher likelihood of interactive effects based on the respective modes of action (e.g. propiconazole with neonicotinoids or FPF) and co-occur in the environment. We provide a simple way to measure synergistic effects on bee behaviour and survival following a standard ecotoxicological test. Our procedure could be implemented fairly easily in pesticide RA procedures, within the LD_50_ toxicity test scheme [60].

## Supplementary Material

ESM Methods and Results

## Data accessibility

Data available from the Dryad Digital Repository: https://doi.org/10.5061/dryad.5f87k5v [83].
