# Supplementary material for: Lethal and sublethal synergistic effects of a new systemic pesticide, flupyradifurone (Sivanto®), on honeybees
Source: Proc Biol Sci. 2019 Apr 10;286(1900):20190433. doi: 10.1098/rspb.2019.0433 (PMC6501679; doi:10.1098/rspb.2019.0433)
Supplement: ESM Methods and Results [file rspb20190433supp1.pdf]

Electronic Supplementary Material (ESM)

Proceedings of the Royal Society B: Biological Sciences

Manuscript ID: RSPB-2019-0433

Doi: 10.1098/rspb.2019.0433

Lethal and sublethal synergistic effects of a new systemic pesticide, flupyradifurone (Sivanto®) on honey bees

S. Tosi[1, 2, *] and J. C. Nieh[1]

[1]Division of Biological Sciences
Section of Ecology, Behavior, and Evolution
University of California San Diego (UCSD)

[2]Epidemiology Unit, European Union Reference Laboratory (EURL) for Honeybee Health
University Paris Est, ANSES (French Agency for Food, Environmental and Occupational Health and Safety) Animal Health Laboratory
Maisons-Alfort, France

*Corresponding author. E-mail: tosi.biology@gmail.com

ESM Methods

This study was conducted at University of California San Diego (UCSD), Division of Biological Sciences (La Jolla, CA, USA). We used six healthy honey bee colonies (*Apis mellifera ligustica* Spinola, 1806, 10 frames per colony [1–3]) housed at the UCSD Biology Field Station apiary. In total, we recorded the survival and abnormal behaviours of 1860 bees and the weight of 354 bees. All assessments were conducted by experimenters blind to the treatments. We followed standard toxicological procedures for pesticide tests on bees [4,5].

Honey bee preparation

Foragers are the bees most likely exposed to flupyradifurone (FPF) since they can directly collect pollen and nectar from treated crops. In-hive bees can also be exposed to FPF because they receive the nectar and pollen collected by the foragers and consume potentially contaminated honey and pollen stores. We captured individually returning pollen foragers at hive entrances in vials. We caught in-hive bees located in combs with brood inside the colonies using standard procedures: these bees were likely nurses [6]. The difference between in-hive and forager bees was additionally confirmed by our weight results (see Results). After collection, in-hive and forager bees were separately placed into plastic cages (11 x 11 x 9 cm, 10 bees/cage) and maintained in an incubator at 25 ± 1°C and 50-80% RH for 72 h [5]. To facilitate consumption of the test solution, the bees were starved for 1 h before feeding [5,6].

Pesticide concentrations and doses

FPF (4D IRAC subgroup) is a newly developed systemic insecticide [7] that was first marketed in 2014 in Guatemala and Honduras [8], then in 2015 in USA [9], EU [10], and other countries [11]. Because FPF is a relatively recent pesticide, there is limited environmental contamination data available [12,13]. Nonetheless, it can be used on diverse crops (vegetables, potatoes, pome fruits, grapes, citrus, cotton, soybean, coffee, cocoa, hops, and ornamentals) though multiple application methods (spray, drip irrigation, soil treatments, and seed treatments) [8,14].

Because foragers mainly consume nectar, we calculated the field-realistic exposure of foragers based on the residue of FPF found in nectar (a realistic carbohydrate source) collected

by a forager, as sampled from its honey stomach [13]. In-hive bees consume nectar and pollen (realistic diet containing carbohydrates and proteins), and we therefore considered nectar and pollen contamination when estimating in-hive bee pesticide intake [15,16].

FPF was found at 4.3 ppm and 4.1 ppm in the nectar in the honey stomachs of bees that were foraging on oilseed rape crops respectively 1 and 3 days after FPF spray treatment (US EPA, 2014). Pollen collected by bees foraging on oilseed rape fields contained 21 ppm of FPF [13]. We simulated a scenario in which bees were foraging on oilseed rape crops, and therefore used FPF residues in nectar (4.3 ppm) and pollen (21 ppm) of oilseed rape. However, bees can be exposed to FPF at even higher concentrations, and for longer periods. Bees ingested FPF when collecting nectar from cotton (22 ppm) or pollen from apple (39 ppm) or blueberry (68 ppm) crops [13]. FPF was found for about 3 weeks in nectar collected by foragers, and up to nearly 5 months after initial exposure in the nectar and honey stored inside bee colonies [13].

With respect to dosages, we calculated the worst-case field-realistic FPF oral exposure levels for bees using European Food Safety Authority (EFSA) and Environmental Protection Agency (EPA) methods. Using these methods, we estimate that foragers collecting nectar in a field previously treated with FPF can be exposed to 550 ng FPF/bee per foraging flight, and up to 5504 ng FPF/bee per foraging day. These calculations were based on EFSA [16] and used the highest field-realistic FPF concentration found in the honey stomachs of bees that were foraging on oilseed rape crops, 4.3 ppm [13], and the sugar concentration of oilseed rape nectar (10% w/w [17,18]). Bees can be exposed to up to 1564 ng FPF/bee/foraging flight when they forage on nectar from cotton fields. These calculations were based on EFSA [16] and used the highest field-realistic FPF concentration found in the honey stomachs of bees that were foraging on cotton crops, 22 ppm [13], and a low field-realistic sugar concentration of cotton nectar (18%, [19]). Unlike foragers, nurses ingest less nectar and more pollen, leading to a field-realistic exposure of 2402 ng FPF/bee/day [16]. This calculation was based on EFSA guidelines [16] and considered intake of FPF contaminated pollen using the highest field-realistic empirical FPF concentration in oilseed rape pollen (21 ppm [13]). According to other calculations [15], the refined Estimated Environmental Concentration (EEC) of FPF is 970 ng/bee and 1256 ng/bee for nurses and foragers, respectively, when colonies forage in oilseed rape crops [13]. When bees forage nectar in cotton fields, refined EEC for workers reaches 6370 ng FPF/bee [13]. Thus, we used a FPF dose of 375 ng/bee that was lower than the field-realistic scenario in which bees

ingested FPF contaminated oilseed nectar during a single foraging flight. The tested FPF dose of 750 ng/bee was less than the field-realistic scenario in which bees ingested contaminated oilseed nectar for 1 day or cotton nectar for a foraging flight.

FPF and propiconazole (PRO) are used in a variety of vegetable, fruit, and ornamental plants that are visited by bees. Because FPF is a new pesticide, no monitoring studies have yet tested its co-occurrence as a contaminant with other pesticides. However, FPF and neonicotinoids are approved for use on many of the same crops [8,13,14,20–22], and pollen collected by bees contained both neonicotinoids and SBI (Sterol Biosynthesis Inhibitor) fungicides [23]. Further screening of environmental contamination following large-scale real world use after widespread commercialization is desirable [24]. The US EPA [13] assessed the combined effect of FPF and the fungicide, tebuconazole, and found that the addition of tebuconazole (ratio 1:7.5, respectively) decreased FPF LD_{50} of in-hive bees by 6 fold (1200 ng FPF/bee vs. 200 ng FPF+tebuconazole/bee). Although risk assessors are developing models to predict multiple chemical interactions based on chemical characteristics, ultimately reducing the amount of laboratory trials needed to assess risk, the data available is still poor and ultimately require empirical validation [25,26].

The acute oral dose-response relationship (i.e. LD_{50} test) was evaluated using five doses in a geometric series, with a common ratio factor of 2 ($FPF_{LD50 \text{ dose range}}$: 750-12000 ng/bee) [5]. Because of the high mortality of the FPF+PRO treatments, we tested an additional lower dose of 375 ng FPF/bee (corresponding to 37.5 ppm), instead of the higher 12000 ng FPF/bee, for the combined treatment. In table 1, we compare FPF+PRO treatments that used 375 ng FPF/bee with a higher dose of FPF alone (750 ng/bee), but our estimate of synergism is likely conservative: as expected 750 ng FPF/bee led to stronger effects than 375 ng FPF/bee (data from preliminary test). Because of high summer mortality, we tested an additional lower dose of DIM (25 ng DIM/bee, corresponding to 25 ppm), instead of the higher 800 ng DIM/bee. The synergistic effects of FPF+PRO were only tested in the summer, since this season is the most standard one for testing toxicity [5].

We used analytical grade FPF (CAS# 951659-40-8, PESTANAL® analytical standard, Sigma-Aldrich, purity: 99.9%), DIM (CAS# 60-51-5, PESTANAL® analytical standard, Sigma-Aldrich, purity: 99.5%), and PRO (CAS# 60207-90-1, PESTANAL® analytical standard, Sigma-Aldrich, purity: 99.2%) to prepare stock solutions respectively containing 3 mg FPF/g

double-distilled H_2O, 1 mg DIM/g double-distilled H_2O, and 100 mg PRO/g acetone [5]. All bees, including control bees, were fed the same amount of solvent (0.7%). The solutions were maintained at 4 °C inside a bottle completely wrapped in aluminum foil to avoid light degradation. The stock solutions were diluted with 1.8 M sucrose solution (corresponding to 50% w/w) to prepare the final solutions that were fed to the bees.

Survival: synergistic and individual effects

Bee mortality was assessed 1 h, 2 h, 4 h, and each 24 h after treatment, up to a maximum of 72 h. A bee was considered dead when was immobile and did not react to any stimulation [4]. The LD_{50} of FPF, DIM, and FPF+PRO were estimated at 48 h after exposure [5]. Because of the very low effect of FPF alone at 375 ng FPF/bee, we only tested this FPF dose in combination with PRO.

Weight assessment

The effects of a given pesticide dose may depend upon bee weight. We therefore measured the weight of 354 pesticide-free bees. Because the amount of food ingested could influence the body weight, the bees were fed the same type of food (50% w/w sucrose solution, *ad libitum*) and frozen at the same time before weighing.

Statistical analysis

We used Fit Proportional Hazards models to separately test the effects of FPF, DIM, or FPF+PRO doses, season (early spring vs. summer), worker type (in-hive vs. forager bees), colony, and all interactions on bee survival (table S5). Because the FPF+PRO treatment was only tested in summer, we used the same model, but without season. Significant effects were further analysed with Kaplan-Meier survival analyses (Wilcoxon Chi-square values) following visual data inspection.

Probit analysis [27] was used to estimate the LD_{50} of FPF, DIM, and FPF+PRO across season (early spring vs. summer) and worker type (in-hive vs. forager bees). Because the treatment FPF+PRO was only tested in summer, this treatment was not tested across seasons. Based on LD_{50} values, we defined biologically significant synergy as mixtures with minimum two-fold difference between observed and predicted effect concentrations using the

Concentration Addition (CA) reference model [28]. We used the CA model because it is recommended for risk assessment purposes [29], and we used the two-fold difference limit to avoid false positives while focusing on synergistic effects of quantitative importance [28]. The ratio between predicted and observed effects of binary mixtures is defined as the Model Deviation Ratio (MDR) [30]. We used the MDR to define if the interaction of the chemical mixture FPF+PRO caused synergistic (MDR > 2), additive ($0.5 \leq MDR \leq 2$), or antagonistic (MDR < 0.5) effects [30]. The MDR was calculated using the Toxic Unit (TU) of the individual pesticides (FPF, PRO) and of the binary chemical mixture (FPF+PRO). The TU is defined as the ratio between the concentration of a mixture component and its toxicological acute (LD_{50}) endpoint [16]. Our TU calculations were based on the LD_{50} of FPF (our data, reported in the Results section; $LD_{50,\text{summer in-hive}}$ = 2995 ng/bee, $LD_{50,\text{summer foragers}}$ = 1865 ng/bee), PRO (LD_{50} > 100000 ng/bee [31]), and FPF+PRO (our data, reported in the Results section; $LD_{50,\text{summer in-hive}}$ = 758 ng/bee, $LD_{50,\text{summer foragers}}$ = 353 ng/bee). We calculated the TU of our chemical mixture (FPF+PRO) as the sum of the TUs of each individual chemical in the mixture.

We used a Mixed Model with a REML algorithm to test the effects of FPF or DIM doses, season (early spring vs. summer), worker type (in-hive vs. forager bees), and all interactions on the frequency of bees exhibiting at least one abnormal behaviour 1 h, 2 h, and 4 h after treatment (table S7-S8). Colony was included as a random factor. Because the FPF+PRO treatment was only tested in summer, we used the same model, but without season. We applied the square root-transformation on the frequency of bees exhibiting an abnormal behaviour. We determined the minimum dose that was significantly different from control using the Least-Square Means contrast test and visual data inspection.

We applied a binomial proportion model [32,33] to test for synergistic effects of FPF and PRO on bee survival (figure 1A-B) and behaviour (figure 3A-B). We used the additive effects model [34], in which synergism is defined as the combined effect of multiple stressors significantly exceeding the sum of effects elicited by individual stressors. The R scripts (p.adjust function) used are available in the electronic supplementary material (ESM), and focused on testing synergistic, not antagonistic, effects. We used a script modified from Tosi *et al.* [33] and Sgolastra *et al*. [32] that tested for synergistic effects by testing if the difference between the observed and the expected effect (either mortality or presence of abnormal behaviour) of the

combined treatment could arise by chance alone or was larger than the simple additive effect of both stressors.

The 0 ng/bee dose treatment was the control for each pesticide. Treatment A consisted of bees exposed only to each specific dose of FPF, for a total of four doses (750, 1500, 3000, 6000 ng/bee). Treatment B consisted of bees only exposed to PRO (7000 ng/bee). Bees exposed to both FPF and PRO (FPF+PRO) were assigned to the combined treatment (AB). We calculated the expected effect proportion of the combined treatment as $P_{ABExp} = P_A + (1-P_A) P_B$, where P_A and P_B are the observed effect proportions in the FPF and PRO treatments, respectively. We used Wald confidence intervals to build a hypothesis test for the difference between two proportions. We separately determined the synergistic effects at each assessment time and used the Holm method to correct for multiple comparisons ($\alpha = 0.05$), as recommended by the R script protocol (ESM). We tested the effects across two worker types (in-hive vs. foragers) during summer. We calculated the Risk Ratio (RR) and the Risk Difference (RD) to quantitatively express the size of the interactive effect of the chemical mixture on bee survival (frequency of dead bees, table S3) and behaviour (frequency of abnormally behaving bees, table S4) [35,36]. The RR was determined by dividing the observed effect by the expected effects (i.e. dividing the cumulative incidence in the exposed group by the cumulative incidence in the unexposed group) and therefore cannot be calculated when the expected effect is 0 [35,36]. To estimate the effect size of the pesticide mixture at all time points after treatment, we calculated the RD, the difference between the ratio of observed and expected effects (i.e. subtracting the cumulative incidence in the unexposed group from the cumulative incidence in the exposed group).

A Mixed Model (REML algorithm) was used to test the effects of season (early spring vs. summer), worker type (in-hive vs. forager bees), and all interactions on bee weight (table S10). Colony was included as a random factor.

Our statistical models were run with R v3.3.2 [37], JMP v10.0 (SAS Statistical Software), and Polo Plus v.2.0 (LeOra Software) software. We used residuals analysis to confirm that our data met parametric assumptions. We report mean ± 1 standard error (s.e.m.), and 95% Confidence Intervals for LD_{50} values [38]. We used an alpha value of 0.05. We applied the Dunn-Sidak method [39] to correct for multiple comparisons when appropriate, and indicated with ^{DS} the corrected statistical tests. We applied stepwise model simplification, building models with all interactions, and then removing them if they were not significant.

ESM Results

Our trials met the official guidelines for toxicity tests [5] because the 24 h LD_{50} of DIM was 114 ng/bee, within the required standard range of 100-350 ng/bee. Moreover, in-hive bees mortality of control treatments was low and within specified limits (≤10%) [5].

FPF doses up to 750 ng/bee caused little mortality (≤10%, not statistically different from control) when we applied the standard toxicological protocol (i.e. using in-hive bees), showing that FPF doses up to 750 ng/bee were sublethal [5].

DIM was more toxic in early spring

There was a significant effect of season on survival of bees exposed to DIM (Fit proportional hazards, $p < 0.0001$, tables S5-S6). DIM was significantly more toxic in early spring, as compared to summer. There were no significant interactions ($p > 0.13$). There was no significant seasonal effect of DIM LD_{50} (figure S2). There was a significant effect of the interaction dose × season or dose × season × worker type on bee abnormal behaviours after treatment with DIM (1-4 h: $p < 0.001$) (table S8).

There was a significant effect of season on bee abnormal behaviours 1 h and 2 h after treatment with DIM (Mixed Model_{REML}, $p < 0.0001$, table S8). There was no significant effect of season on bee behaviours at 4 h after treatment of DIM ($p > 0.62$).

Bee weight varied depending on worker type and season

There was a significant effect of worker type ($F_{1,351} = 44.66$, $p < 0.0001$) and season ($F_{1,350} = 5.58$, $p = 0.019$) on bee weight (table S10). In-hive bees were significantly heavier than foragers (+11%), and summer bees were significantly heavier than early spring bees (+5%). There was no significant effect of the interaction worker type × season on pesticide-free bee weight ($p > 0.59$). The effect of colony accounted for 1% of model variance.

ESM Discussion

FPF and DIM showed opposite effects across season

Season consistently influenced pesticide toxicity (figures 2, 4, S2-S4), but its effect varied depending on the active ingredient. Bees were more susceptible to FPF in summer, while bees exposed to DIM were more affected in early spring (ESM). This variability is reflected in the results of prior studies, although we provide the first results showing how this variability can occur even when testing bees from the same apiary during the same seasons. Summer bees are typically more sensitive to pesticides as compared to winter bees [40–43]. However, the neonicotinoid imidacloprid reduced survival of winter bees, as compared to summer bees [41]. In early spring (first few weeks after overwintering), bees were more susceptible to the neonicotinoids, clothianidin and thiamethoxam, than summer bees [44]. The physiological modifications that bees experience across seasons, including variations in midgut structure (which can be a barrier to chemical transfer) and target receptor density, may account for some seasonal variability in pesticide toxicity [42,44]. Interestingly, the transition in bee sensitivity to pesticide seems to occur between early and late spring [44].

ESM tables

Table S1. Definitions of abnormal behaviours exhibited by bees. We provide a video showing examples of abnormal behaviours in the ESM, and additional details are in table S2.

Name	Definition
Motion coordination deficits	Loss of coordination consisting of falling or stumbling while walking, walking in circles, walking and flying with erratic and irregular movements, and bees that flap their wings while upside down.
Hyperactivity	Excitation manifested as rapid walking, sometimes including short jumps and flight attempts, fast movements of legs and antennae.
Apathy	Hypoactivity consisting of remaining largely motionless or walking very slowly. Such bees also have severely reduced or delayed reactions to stimulation provided by light, movements of other bees, or air currents (e.g. generated by nearby bees).
Curved-down abdomen	The abdomen is unnaturally curved and is flexed ventrally, cramps.
Moribund	The bee appears close to death and exhibits partial paralysis with slight movements of legs and antennae. Will respond slightly to mechanical stimulation.

Table S2. List of abnormal behaviours observed in the videos recorded during preliminary ecotoxicological trials to highlight and determine the types of common abnormal bee behaviours following pesticide consumption in sucrose solution. The video is available in the ESM (below) and Dryad Digital Repository, and further details are in table S1.

Video ID	Abnormal behaviour type	Description of abnormal behaviour(s)
1	Motion coordination deficits, hyperactivity	One bee shows hyperactivity, loss of coordination, stumbling, and erratic and irregular walking and flight movements.
2	Motion coordination deficits, hyperactivity	One bee is lying on the floor and shows loss of coordination with rapid twitching of legs and wings without flying for prolonged time.
3	Motion coordination deficits	One bee lies on the floor and shows rapid twitching of legs and wings without flying for prolonged time, loss of coordination.
4	Motion coordination deficits, hyperactivity	One bee shows hyperactivity, loss of coordination, stumbles, moves with erratic and irregular movements: atypical circular patterns.
5	Motion coordination deficits, hyperactivity	One bee lies on the floor and shows rapid twitching of legs and antennae, loss of coordination. The bees were recorded soon after exposure (acute oral) to the lower field-realistic dose of FPF (insecticide) and the sublethal dose of PRO (SBI fungicide; this fungicide dose alone caused no abnormal behaviours).
6	Hyperactivity	One bee (bottom, left) shows excitation and rapid movements of legs and antennae.
7	Apathy	Two hypoactive bees on the right side of the video.
8	Apathy	One hypoactive bee on the right side of the cage.
9	Curved-down abdomen, motion coordination deficits	One bee, standing next to the transparent cage door, showing a curved-down abdomen, and exhibiting irregular movements. Behind it, a bee rapidly twitches its legs and is unable to stand. On the left, moribund bees might seem dead but they exhibit partial paralysis and show slight movements of legs and antennae. This video shows a preliminary trial with bees exposed to the lower field-realistic dose of FPF and the sublethal dose of PRO (SBI fungicide) rapidly after acute oral treatment.
10	Moribund	One bee (close to cage front door) showing only slight movements of its legs and antennae, is unable to stand and appears close to death.

Table S3. Synergistic effects of FPF+PRO on bee survival, depending on FPF dose, worker type, and time after exposure (1-48 h). We show the Risk Ratio (RR, observed/expected), the Risk Difference (RD, observed-expected), and the statistical results (binomial proportion test, Holm corrected, *N* = 420). We report “NA” when the expected or observed mortality was 0. In this R script analysis, we only test for synergism.

Worker type	FPF dose (ng/bee)	PRO dose (ng/bee)	RR after treatment (h)					RD after treatment (h)					*P*-value after treatment (h)				
			1	2	4	24	48	1	2	4	24	48	1	2	4	24	48
In-hive	0	7000	NA	NA	NA	3.0	0.4	0	0	0	7	-15	1.000	1.000	1.000	0.741	1.000
	750		NA	8.0	9.0	5.5	2.0	23	23	27	44	29	**0.005**	**0.007**	**0.005**	**<0.001**	**0.013**
	1500		NA	4.5	3.3	3.3	2.0	23	23	30	37	29	**0.005**	**0.014**	**0.009**	**0.003**	**0.014**
	3000		4.5	3.0	0.9	1.3	1.3	23	20	-3	19	16	**0.036**	0.091	0.605	0.206	0.253
	6000		1.7	2.2	0.7	0.9	0.9	13	23	-20	-10	-7	0.475	0.112	1.000	1.000	1.000
Forager	0	7000	NA	NA	NA	0.2	0.3	0	0	-7	-27	-46	1.000	1.000	1.000	1.000	1.000
	750		NA	NA	NA	4.9	1.7	20	40	57	64	34	**0.006**	**<0.001**	**<0.001**	**<0.001**	**0.006**
	1500		5.0	1.4	1.5	1.4	1.2	27	10	17	19	13	**0.015**	0.387	0.351	0.351	0.387
	3000		11.0	2.6	1.4	1.3	1.1	33	27	20	22	11	**0.001**	**0.037**	0.107	0.107	0.202
	6000		1.0	1.2	0.8	1.0	1.0	0	7	-13	0	0	1.000	1.000	1.000	1.000	1.000

Table S4. Synergistic effects of FPF+PRO on the frequency of abnormal behaviours, depending on FPF dose, worker type, and time after exposure (1-4 h). We show the Risk Ratio (RR, observed/expected), the Risk Difference (RD, observed-expected), and the statistical results (binomial proportion test, Holm corrected, N = 420). We report “NA” when the expected or observed mortality was 0.

Worker type	FPF dose (ng/bee)	PRO dose (ng/bee)	RR after treatment (h)			RD after treatment (h)			*P*-value after treatment (h)		
			**1**	**2**	**4**	**1**	**2**	**4**	**1**	**2**	**4**
In-hive	0	7000	NA	NA	NA	0	0	0	1.000	1.000	1.000
	750		7.3	7.3	10.0	63	63	60	**<0.001**	**<0.001**	**<0.001**
	1500		1.7	2.3	2.3	23	33	27	**0.031**	**0.009**	**0.022**
	3000		0.8	1.0	1.6	-13	-3	23	1.000	1.000	0.094
	6000		1.0	0.7	2.0	-3	-27	27	1.000	1.000	**0.043**
Foragers	0	7000	NA	NA	NA	-7	-7	-7	1.000	1.000	1.000
	750		2.5	15.0	5.5	30	47	30	**0.005**	**<0.001**	**0.002**
	1500		0.7	1.9	2.3	-17	30	27	0.906	**0.022**	**0.022**
	3000		0.7	1.2	1.3	-20	10	7	0.954	0.654	0.654
	6000		1.8	1.1	0.7	30	7	-13	**0.021**	0.604	0.860

Table S5. Effect of dose, season, worker type, and all interactions on bee survival after exposure to FPF (DF_{Model} = 20), FPF+PRO (DF_{Model} = 8), or DIM (DF_{Model} = 10) (Fit proportional hazard). We report in bold the significant effects. For each factor, we report the statistical values of the last stepwise model simplification that included the factor.

Active ingredient	Factor	DF	L-R χ^2	*P*-value
FPF	Dose	5	297.74	**<0.0001**
	Season	1	29.11	**<0.0001**
	Worker type	1	49.60	**<0.0001**
	Dose × Season	5	14.98	**0.0105**
	Dose × Worker type	5	21.85	**0.0006**
	Season × Worker type	1	17.40	**<0.0001**
	Dose × Season × Worker type	5	4.90	0.4277
	Colony	2	5.86	0.0535
DIM	Dose	6	300.10	**<0.0001**
	Season	1	17.54	**<0.0001**
	Worker type	1	37.89	**<0.0001**
	Dose × Season	4	3.82	0.4306
	Dose × Worker type	6	5.76	0.4511
	Season × Worker type	1	2.25	0.1332
	Dose × Season × Worker type	5	2.69	0.6112
	Colony	2	0.22	0.8946
FPF+PRO	Dose	5	103.29	**<0.0001**
	Worker type	1	12.76	**0.0004**
	Dose × Worker type	5	1.32	0.9328
	Colony	2	3.15	0.2071

Table S6. Effect of dose, season, and worker type on bee survival, depending on FPF and DIM doses (Kaplan-Meier[DS], Wilcoxon). We report in bold the significant effects after Dunn-Sidak correction for multiple comparisons (FPF: dose and season, k = 4, adjusted α = 0.0127; worker type: k = 5, adjusted α = 0.0102; DIM: dose, k = 6, adjusted α = 0.0085; season, k = 4, adjusted α = 0.0127; worker type: k = 5, adjusted α = 0.0102). For the dose effect, we compared all doses with control. For the seasonal effect, we did not use 25 and 800 ng DIM/bee in both seasons, since in summer we tested 25 ng DIM/bee instead of 800 ng DIM/bee because using the higher dose of DIM would have resulted in excessively high mortality precluding using this data ("NA"). We report the values of tested comparisons only.

		Dose effect			Season effect			Worker type effect		
**Dose (ng/bee)**		**χ^2**	**DF**	***P*-value**	**χ^2**	**DF**	***P*-value**	**χ^2**	**DF**	***P*-value**
**Control**	**0**		NA					21.97	1	**<0.0001**
**FPF**	**750**									
	**1500**	3.89	1	**0.0487**	2.96	1	0.0855	14.29	1	**0.0002**
	**3000**	47.26	1	**<0.0001**	6.22	1	**0.0127**	18.48	1	**<0.0001**
	**6000**	115.97	1	**<0.0001**	13.23	1	**0.0003**	7.70	1	**0.0055**
	**12000**	155.89	1	**<0.0001**	0.08	1	0.7802	1.25	1	0.2629
**DIM**	**25**	8.11	1	**0.0044**		NA				
	**50**	10.17	1	**0.0014**	6.32	1	**0.012**	11.49	1	**0.0007**
	**100**	47.82	1	**<0.0001**	1.84	1	0.1744	17.47	1	**<0.0001**
	**200**	130.64	1	**<0.0001**	9.35	1	**0.0022**	9.38	1	**0.0022**
	**400**	153.32	1	**<0.0001**	16.99	1	**<0.0001**	15.76	1	**<0.0001**
	**800**	156.53	1	**<0.0001**		NA		4.80	1	**0.0285**

Table S7. Effect of FPF or FPF+PRO dose on the frequency of bees exhibiting at least one abnormal behaviour. Based on visual inspection of the data, we made limited tests of the effect of each pesticide dose as compared to control treatments (Mixed Model$_{REML}$, Contrast test^{DS}). We report only tested comparisons.

Active ingredient (name)	Time from treatment (h)	FPF dose (ng/bee)	DF numerator	DF denominator	*F* Ratio	*P*-value
FPF	1	750	1	52	6.96	**0.0109**
		1500	1	52	106.60	**<0.0001**
		3000	1	52	182.58	**<0.0001**
		6000	1	52	193.95	**<0.0001**
		12000	1	52	232.27	**<0.0001**
	2	1500	1	62	24.41	**<0.0001**
		3000	1	62	42.44	**<0.0001**
		6000	1	62	56.77	**<0.0001**
		12000	1	62	67.75	**<0.0001**
	4	1500	1	60	16.07	**0.0002**
		3000	1	60	32.51	**<0.0001**
		6000	1	60	46.74	**<0.0001**
		12000	1	60	18.98	**<0.0001**
FPF+PRO	1	375	1	32	164.81	**<0.0001**
		750	1	32	176.36	**<0.0001**
		1500	1	32	145.40	**<0.0001**
		3000	1	32	176.68	**<0.0001**
		6000	1	32	194.62	**<0.0001**
	2	375	1	32	228.32	**<0.0001**
		750	1	32	276.09	**<0.0001**
		1500	1	32	279.59	**<0.0001**
		3000	1	32	279.07	**<0.0001**
		6000	1	32	241.88	**<0.0001**
	4	375	1	25	148.24	**<0.0001**
		750	1	25	166.23	**<0.0001**
		1500	1	25	157.22	**<0.0001**
		3000	1	25	146.20	**<0.0001**
		6000	1	25	133.70	**<0.0001**

Table S8. Abnormal behaviours of bees exposed to FPF, FPF+PRO, and DIM, depending on season, worker type, doses, and time after exposure. The effect of FPF+PRO was not tested across season. We tested the effects up to 4 h after treatment because the high mortality elicited by the pesticides at 24-48 h limited the sample size and precluded a rigorous analysis of abnormal behaviours.

Active ingredient	Time after treatment (h)	Colony effect (%)	Factor	DF numerator	DF denominator	*F* Ratio	*P*-value
FPF	1	5	Dose	5	52	82.54	**<0.0001**
			Season	1	52	25.74	**<0.0001**
			Worker type	1	52	4.16	**0.0464**
			Dose × Season	5	52	5.43	**0.0004**
			Dose × Worker type	5	52	4.90	**0.0010**
			Season × Worker type	1	46	1.61	0.2115
			Dose × Season × Worker type	5	46	2.25	0.0654
	2	<1	Dose	5	62	25.60	**<0.0001**
			Season	1	62	7.74	**0.0071**
			Worker type	1	62	0.13	0.7155
			Dose × Season	5	46	0.25	0.9354
			Dose × Worker type	5	46	2.44	**0.0486**
			Season × Worker type	1	46	2.75	0.1041
			Dose × Season × Worker type	5	46	2.40	0.0515
	4	1	Dose	5	60	14.99	**<0.0001**
			Season	1	60	0.43	0.5161
			Worker type	1	60	0.31	0.5798
			Dose × Season	5	44	0.71	0.6221
			Dose × Worker type	5	44	1.15	0.3499
			Season × Worker type	1	44	0.08	0.7759
			Dose × Season × Worker type	5	44	1.48	0.2144

Active ingredient	Time after treatment (h)	Colony effect (%)	Factor	DF numerator	DF denominator	*F* Ratio	*P*-value
DIM	1	<1	Dose	5	47	19.68	**<0.0001**
			Season	1	47	31.71	**<0.0001**
			Worker type	1	47	27.97	**<0.0001**
			Dose × Season	5	47	5.24	**0.0007**
			Dose × Worker type	5	47	2.84	**0.0254**
			Season × Worker type	1	46	2.28	0.1382
			Dose × Season × Worker type	5	47	2.97	**0.0208**
	2	<1	Dose	5	52	81.00	**<0.0001**
			Season	1	52	44.43	**<0.0001**
			Worker type	1	52	8.65	**0.0049**
			Dose × Season	5	46	1.15	0.3501
			Dose × Worker type	5	52	5.43	**0.0004**
			Season × Worker type	1	46	0.36	0.5515
			Dose × Season × Worker type	5	52	5.23	**0.0006**
	4	2	Dose	5	44	45.40	**<0.0001**
			Season	1	44	0.24	0.6241
			Worker type	1	44	0.08	0.7775
			Dose × Season	5	44	5.06	**0.0009**
			Dose × Worker type	5	44	8.90	**0.0000**
			Season × Worker type	1	44	4.87	**0.0325**
			Dose × Season × Worker type	5	44	5.05	**0.0010**
FPF+PRO	1	<1	Dose	6	32	82.17	**<0.0001**
			Worker type	1	32	1.09	0.3052
			Dose × Worker type	6	26	0.68	0.6662
	2	<1	Dose	6	32	124.83	**<0.0001**
			Worker type	1	32	0.24	0.6289
			Dose × Worker type	6	26	1.35	0.2714
	4	28	Dose	6	25	72.59	**<0.0001**
			Worker type	1	25	4.00	0.0565
			Dose × Worker type	6	25	4.56	**0.0030**

Table S9. The effects of interactions dose × worker type and dose × season on the abnormal behaviours of bees depending on FPF or FPF+PRO exposure. Based upon visual inspection of the data, we conducted limited tests of the effect of worker type (in-hive vs. forager bees) or season (early spring vs. summer) at each specific dose treatment (Mixed Model$_{REML}$, Contrast test^{DS}). The effect of FPF+PRO was not tested across season. We report only tested comparisons.

Effect	Active ingredient (name)	Time from treatment (h)	FPF dose (ng/bee)	DF numerator	DF denominator	*F* Ratio	*P*-value
Dose × Worker type	FPF	1	1500	1	52	17.16	**0.0001**
	FPF	1	3000	1	52	3.35	0.0729
	FPF+PRO	4	375	1	25	9.09	**0.0058**
Dose × Season	FPF	1	750	1	52	26.86	**<0.0001**
	FPF	1	1500	1	52	6.73	**0.0123**
	FPF	1	3000	1	52	13.96	**0.0005**

Table S10. Effect of season, worker type, and their interaction on bee weight. For each factor, we report the statistical values of the latest possible stepwise model simplification (Mixed $Model_{REML}$).

Factor	N	R^2	Colony effect (%)	DF numerator	DF denominator	*F* Ratio	*P*-value
Season				1	350	5.58	**0.0187**
Worker type	354	0.15	1	1	351	44.66	**<0.0001**
Season × Worker type				1	350	0.28	0.5974

ESM figures

Figure S1. The LD_{50} (48 h) of bees exposed to FPF (left and centre) and FPF+PRO (right) across seasons (early spring vs. summer) and worker types (in-hive bees vs. foragers). Above each bar, we show the LD_{50} values. Different letters indicate significant differences. We show the 24 h LD_{50} of summer foragers (light grey bars), because high summer forager mortality at 48 h prevented the accurate estimation of their 48 h LD_{50} (standard LD_{50} estimation time, dark grey bars). Error bars represent 95% confidence intervals ($N_{overall}$ = 1080).

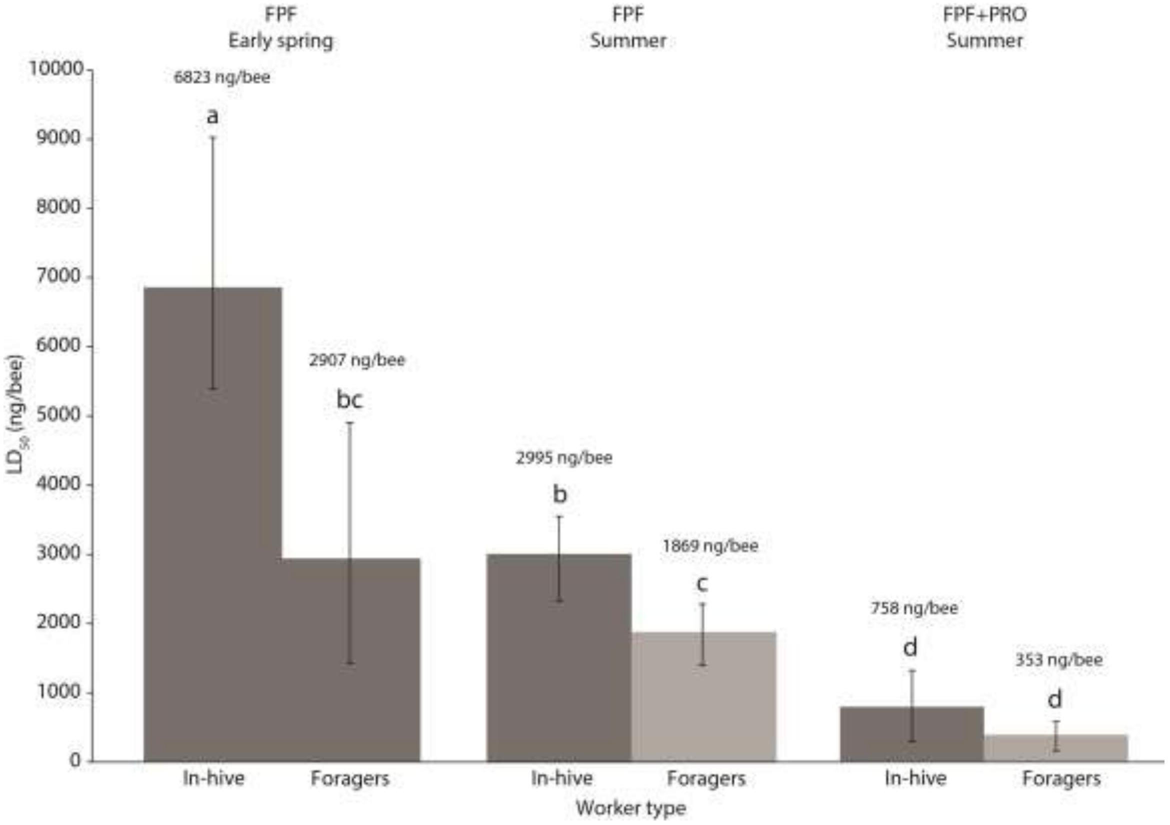

Figure S2. The LD_{50} (48 h) of bees exposed to DIM across seasons (early spring vs. summer) and worker types (in-hive bees vs. foragers). Above each bar, we show the LD_{50} values. Different letters indicate significant differences. We show the 24 h LD_{50} of summer foragers (light gray bars), because high forager mortality of summer foragers at 48 h prevented the accurate estimation of their 48 h LD_{50}. Error bars represent 95% confidence intervals.

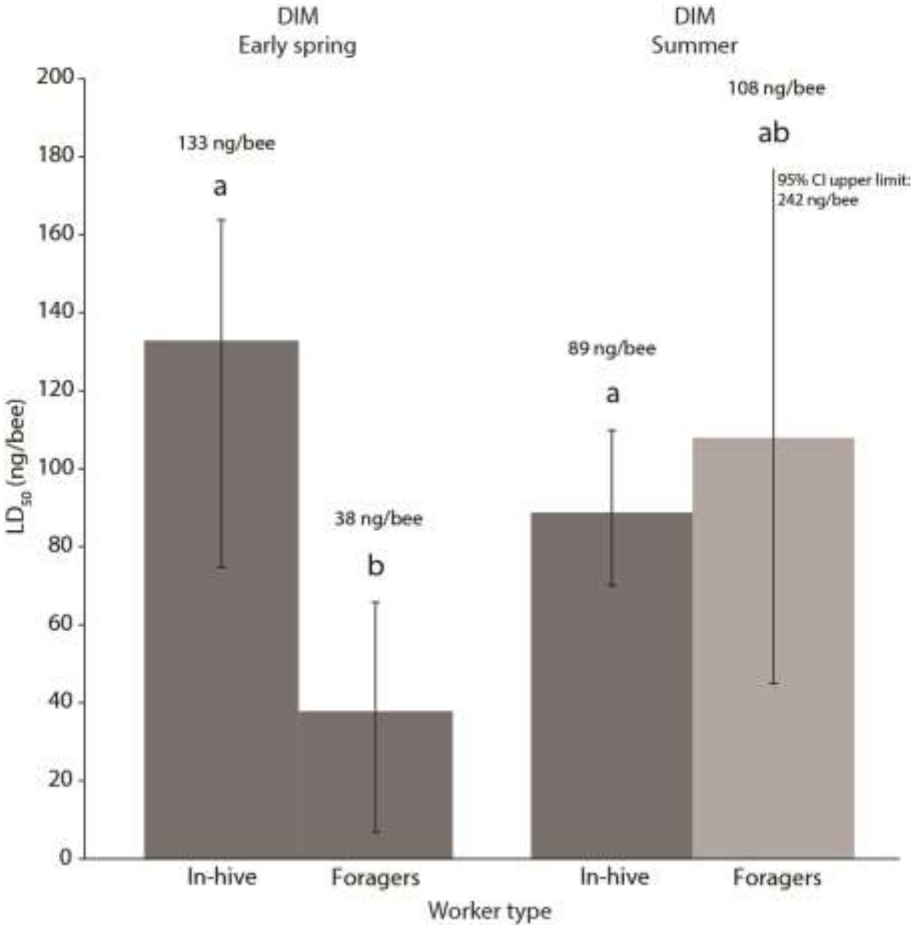

Figure S3. Main effects of (A, D) dose, (B, E) season, and (C, F) worker type on survival of bees exposed to (A, B, C) FPF or (D, E, F) DIM. Asterisks indicate significant differences (Kaplan-Meier[DS], *$p < 0.05$, **$p < 0.01$, **** $p < 0.0001$, table S6). In A and D, we made limited pairwise comparisons testing the dose effect (0-5 dose levels, corresponding to a control dose and between 750-12000 ng FPF/bee in A, 50-800 ng DIM/bee in D) comparing each dose to control based upon visual inspection of the data (Dunn-Sidak corrected, table S6).

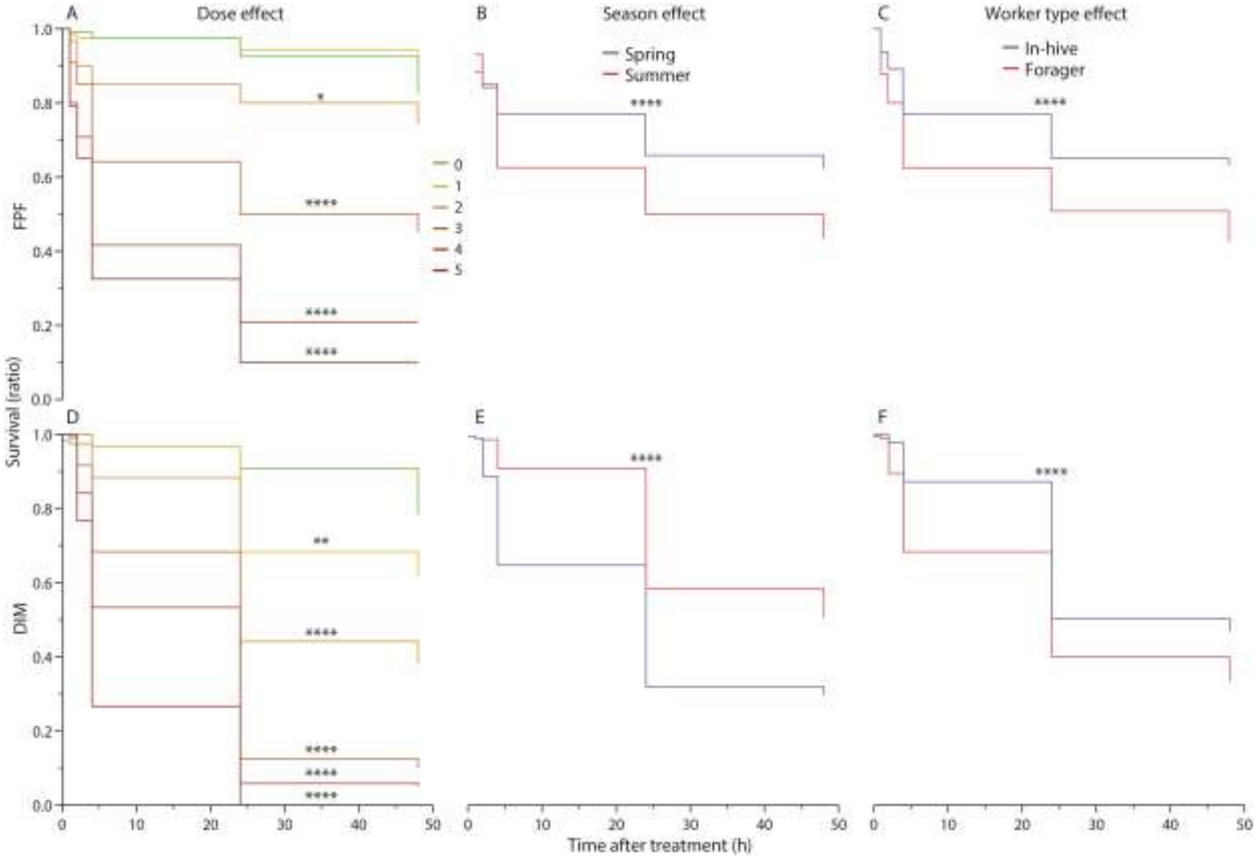

Figure S4. Effect of (A) season and (B, C) worker type on the frequency of bees exhibiting abnormal behaviours after exposure to (A, B) FPF or (C) FPF+PRO doses (0-6 dose levels, corresponding to a control dose and between 375-12000 ng FPF/bee). In figure 3C-D of the main text, these results were pooled by worker type, but are here split by worker type to provide further information. Asterisks indicate significant differences (Mixed Model$_{REML}$, Contrast test^{DS}, *p < 0.05, ***p* < 0.01, ****p* < 0.001, *****p* < 0.0001). We compared the effect of season and worker type within each pesticide dose based on visual estimation (Dunn-Sidak corrected). Main effects and further statistical details are reported in tables S8-S9.

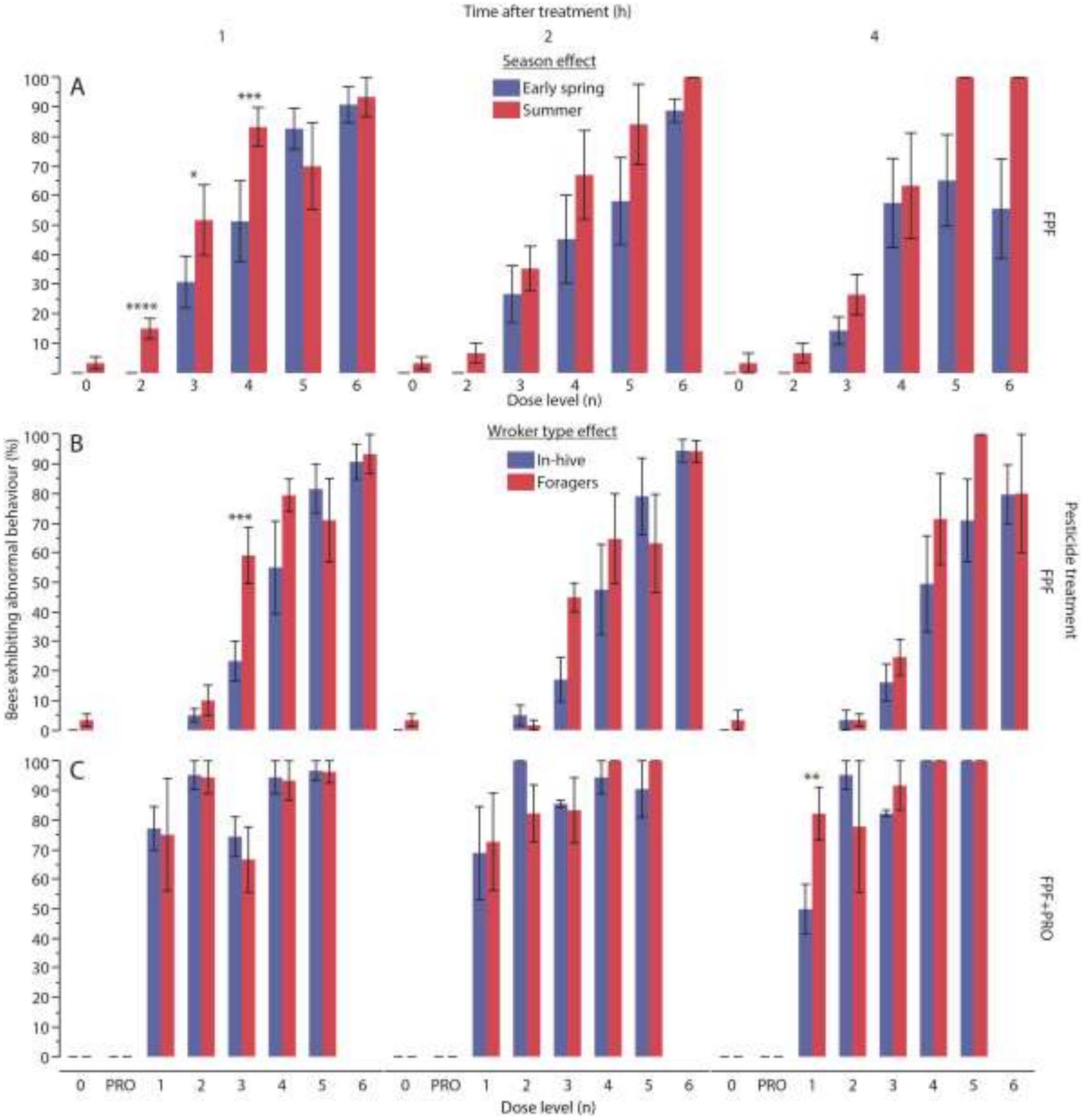

ESM videos

Abnormal behaviours observed in videos recorded during preliminary ecotoxicological trials. The videos highlight and better define the types of common abnormal bee behaviours occurring after oral pesticide consumption in sucrose solution. Further details are available in table S1-S2. The video is available in:

- ✓ the Dryad Digital Repository (https://doi.org/10.5061/dryad.5f87k5v)
- ✓ at this YouTube link: YouTube Video of Abnormal Behaviours
- ✓ at this QR code:

R scripts

R script for testing synergistic effects on survival

```
################################################################################
# Testing the synergistic effects of two chemicals on survival
# Testing for additivity:
#     Confidence interval for binomial proportion difference under Bliss independence.
#
# INPUTS:
# ndead = vector with 3 elements, containing number of dead individuals under
#         treatment A, B and combined.
# ntot  = vector with 3 elements, containing total number of individuals under
#         the 3 treatments.
# p.signif = significance level (usually 0.05).
# alternative = character string specifying the alternative hypothesis.
#
# OUTPUTS:
# See Tosi et al. 2019
################################################################################

ci.bliss.additivity <- function(ndead,ntot,p.signif=0.05,alternative="greater") {
  if (alternative=="two.sided") p.signif <- p.signif/2  # Two-tailed test.
  ndead <- unname(ndead)
  ntot <- unname(ntot)
  p <- ndead/ntot
  pa <- p[1]
  pb <- p[2]
  pab.obs <- p[3]
  vara <- p[1]*(1-p[1])/ntot[1]
  varb <- p[2]*(1-p[2])/ntot[2]
  varab.obs <- p[3]*(1-p[3])/ntot[3]
```

```
  pab.exp <- pa+pb-pa*pb
  varab.exp <- vara+varb+pb^2*vara+pa^2*varb    # Derived with the Delta method.
  p.dif <- pab.obs-pab.exp
  sd.all <- sqrt(varab.obs+varab.exp)
  z <- qnorm(1-p.signif)
  out <- list(pA=pa,pB=pb,pAB.obs=pab.obs,pAB.exp=pab.exp,p.Dif=p.dif,
              VarA=vara,VarB=varb,VarAB.obs=varab.obs,VarAB.exp=varab.exp,Var.All=sd.all^2,
              CI=switch(alternative,
                        two.sided=c(lower=p.dif-z*sd.all,upper=p.dif+z*sd.all),
                        less=c(upper=p.dif+z*sd.all),
                        greater=c(lower=p.dif-z*sd.all)))
  return(out)
}

# Calculates the exact p-value by inverting the hypothesis test.
invert.hypothesis.bliss <- function(n.mort,n.total) {
  fbliss <- function(signif)
ci.bliss.additivity(n.mort,n.total,signif,alternative="greater")$CI["lower"]
  loglik <- function(signif) abs(fbliss(signif))
  return(optimize(loglik,interval=c(0,1),maximum=F,tol=1e-32)$minimum)
}

# Mortality data. Column 1 (e.g. datamort[[1]][,1]) contains the total number of individuals,
labelled "N".
datamort <- list()
datamort[[1]] <- cbind(c(30,30,30),c(0,0,0),c(0,0,0),c(2,0,0),c(7,4,2),c(13,10,5)) # forager, 0
datamort[[2]] <- cbind(c(30,30,30),c(0,0,6),c(0,0,12),c(0,0,17),c(1,4,24),c(7,10,25)) # forager,
750
datamort[[3]] <- cbind(c(30,30,30),c(2,0,10),c(7,0,10),c(10,0,15),c(13,4,21),c(15,10,24)) #
forager, 1500
```

```
datamort[[4]] <- cbind(c(30,30,30),c(1,0,11),c(5,0,13),c(16,0,22),c(20,4,28),c(25,10,30)) # forager, 3000
datamort[[5]] <- cbind(c(30,30,30),c(8,0,8),c(11,0,13),c(25,0,21),c(30,4,30),c(30,10,30)) # forager, 6000
datamort[[6]] <- cbind(c(30,30,30),c(0,0,0),c(0,0,0),c(0,0,0),c(0,1,3),c(3,5,3)) # in-hive, 0
datamort[[7]] <- cbind(c(30,30,30),c(0,0,7),c(1,0,8),c(1,0,9),c(2,1,16),c(4,5,17)) # in-hive, 750
datamort[[8]] <- cbind(c(30,30,30),c(0,0,7),c(2,0,9),c(4,0,13),c(4,1,16),c(4,5,17)) # in-hive, 1500
datamort[[9]] <- cbind(c(30,30,30),c(2,0,9),c(3,0,9),c(12,0,11),c(17,1,23),c(17,5,24)) # in-hive, 3000
datamort[[10]] <- cbind(c(30,30,30),c(6,0,10),c(6,0,13),c(22,0,16),c(29,1,26),c(29,5,27)) # in-hive, 6000

for (i in 1:10) rownames(datamort[[i]]) <- c("TREAT.A","TREAT.B","TREAT.AB") # TREAT.A = FPF; TREAT.B = PRO; TREAT.AB = FPF+PRO
for (i in 1:10) colnames(datamort[[i]]) <- c("N","1h","2h","4h","24h","48h")

cat("------------------------------------------------------------------\n")

# Testing Bliss additivity. All we need to do is to define "n.total" and "n.mort", and then feed invert.hypothesis.bliss() with those two numbers.
# Index i runs from 1 to the number of synergies tested (=1).
# For a generic dataset with 1 endpoint and where nt=total number of individuals and nd=number of dead individuals, we would do: p <- invert.hypothesis.bliss(nt,nd)

for (i in 1:10) {
  a <- datamort[[i]]
  b <- a[,-1]
  p.value <- NULL

# For each endpoint j we test the Bliss hypothesis.
  for (j in 1:5) {
```

```
    n.total <- a[c(1,2,3),1]    # Total number of individuals
    n.mort <- a[c(1,2,3),j+1]   # Number of dead individuals.
    p <- invert.hypothesis.bliss(n.mort,n.total)  # p-value from inverting the hypothesis test.
    p.value <- c(p.value,p)
  }
# Control for multiple comparison, Holm methodology. For cases where there is only 1 endpoint this is obviously not needed.
  p.correct <- p.adjust(p.value,method="holm")

# Formatted output.
  name.data <- c("forager, 0","forager, 750","forager, 1500","forager, 3000","forager, 6000","in-hive, 0","in-hive, 750","in-hive, 1500","in-hive, 3000","in-hive, 6000")
  cat(paste(name.data[i],"\n",sep=""))
  names(p.correct) <- c("1h","2h","4h","24h","48h")
  print(datamort[[i]])
  cat("\n")
  cat(paste(name.data[i],". Observed and expected binomial proportions.\n",sep=""))
  pab <- a[,-1]/a[,1]
  pab <- rbind(pab,pab[1,]+pab[2,]-pab[1,]*pab[2,])
  rownames(pab) <- c("TREAT.A","TREAT.B","TREAT.AB","Expected")
  print(pab)
  cat("\n")
  cat(paste(name.data[i],". Control of type I errors (Holm method) in binomial proportion test.\n",sep=""))
  print(p.correct)

  cat("-----------------------------------------------------------------\n")
}
```

R script for testing synergistic effects on abnormal behaviours

```
##############################################################################
```

```
# Testing the synergistic effects of two chemicals on abnormal behaviours
# Testing for additivity:
#     Confidence interval for binomial proportion difference under Bliss independence.
#
# INPUTS:
# nabnbe = vector with 3 elements, containing number of individuals exhibiting abnormal
#         behaviour under treatment A, B and combined.
# ntot  = vector with 3 elements, containing total number of individuals under
#         the 3 treatments.
# p.signif = significance level (usually 0.05).
# alternative = character string specifying the alternative hypothesis.
#
# OUTPUTS:
# See Tosi et al. 2019
################################################################################

ci.bliss.additivity <- function(nabnbe,ntot,p.signif=0.05,alternative="greater") {
  if (alternative=="two.sided") p.signif <- p.signif/2  # Two-tailed test.
  nabnbe <- unname(nabnbe)
  ntot <- unname(ntot)
  p <- nabnbe/ntot
  pa <- p[1]
  pb <- p[2]
  pab.obs <- p[3]
  vara <- p[1]*(1-p[1])/ntot[1]
  varb <- p[2]*(1-p[2])/ntot[2]
  varab.obs <- p[3]*(1-p[3])/ntot[3]
  pab.exp <- pa+pb-pa*pb
  varab.exp <- vara+varb+pb^2*vara+pa^2*varb    # Derived with the Delta method.
  p.dif <- pab.obs-pab.exp
  sd.all <- sqrt(varab.obs+varab.exp)
```

```
  z <- qnorm(1-p.signif)
  out <- list(pA=pa,pB=pb,pAB.obs=pab.obs,pAB.exp=pab.exp,p.Dif=p.dif,
              VarA=vara,VarB=varb,VarAB.obs=varab.obs,VarAB.exp=varab.exp,Var.All=sd.all^2,
              CI=switch(alternative,
                        two.sided=c(lower=p.dif-z*sd.all,upper=p.dif+z*sd.all),
                        less=c(upper=p.dif+z*sd.all),
                        greater=c(lower=p.dif-z*sd.all)))
  return(out)
}

# Calculates the exact p-value by inverting the hypothesis test.
invert.hypothesis.bliss <- function(n.abnbe,n.total) {
  fbliss <- function(signif)
ci.bliss.additivity(n.abnbe,n.total,signif,alternative="greater")$CI["lower"]
  loglik <- function(signif) abs(fbliss(signif))
  return(optimize(loglik,interval=c(0,1),maximum=F,tol=1e-32)$minimum)
}

# Abnormal behaviour data (individuals exhibiting the behaviour).
# Column 1 (e.g. dataabnbe[[1]][,1]) contains the total number of individuals, labelled "N".
dataabnbe <- list()

dataabnbe[[1]] <- cbind(c(30,30,30),c(0,0,0),c(0,0,0),c(0,0,0)) # in-hive, 0
dataabnbe[[2]] <- cbind(c(30,30,30),c(3,0,22),c(3,0,22),c(2,0,20)) # in-hive, 750
dataabnbe[[3]] <- cbind(c(30,30,30),c(10,0,17),c(8,0,18),c(6,0,14)) # in-hive, 1500
dataabnbe[[4]] <- cbind(c(30,30,30),c(24,0,20),c(21,0,20),c(12,0,19)) # in-hive, 3000
dataabnbe[[5]] <- cbind(c(30,30,30),c(21,0,20),c(23,0,15),c(8,0,16)) # in-hive, 6000
dataabnbe[[6]] <- cbind(c(30,30,30),c(2,0,0),c(2,0,0),c(2,0,0)) # foragers, 0
dataabnbe[[7]] <- cbind(c(30,30,30),c(6,0,15),c(1,0,15),c(2,0,11)) # foragers, 750
dataabnbe[[8]] <- cbind(c(30,30,30),c(19,0,14),c(10,0,19),c(6,0,14)) # foragers, 1500
dataabnbe[[9]] <- cbind(c(30,30,30),c(23,0,17),c(14,0,17),c(6,0,8)) # foragers, 3000
```

```
dataabnbe[[10]] <- cbind(c(30,30,30),c(12,0,21),c(15,0,17),c(13,0,9)) # foragers, 6000

for (i in 1:10) rownames(dataabnbe[[i]]) <- c("TREAT.A","TREAT.B","TREAT.AB") #
TREAT.A = FPF; TREAT.B = PRO; TREAT.AB = FPF+PRO
for (i in 1:10) colnames(dataabnbe[[i]]) <- c("N","1h","2h","4h")

cat("------------------------------------------------------------------\n")

# Testing Bliss additivity. All we need to do is to define "n.total" and "n.abnbe",
#                          and then feed invert.hypothesis.bliss() with those two numbers.
# Index i runs from 1 to the number of synergies tested.
# For a generic dataset with 1 endpoint and where nt=total number of individuals
#                          and nab=number of individuals exhibiting abnormal behaviour,
#                                  we would do: p <- invert.hypothesis.bliss(nt,nab)

for (i in 1:10) {
  a <- dataabnbe[[i]]
  b <- a[,-1]
  p.value <- NULL

# For each endpoint j we test the Bliss hypothesis. J is the # of time assessments
  for (j in 1:3) {
    n.total <- a[c(1,2,3),1]    # Total number of individuals
    n.abnbe <- a[c(1,2,3),j+1]   # Number of bees exhibiting abnormal behaviour.
    p <- invert.hypothesis.bliss(n.abnbe,n.total)  # p-value from inverting the hypothesis test.
    p.value <- c(p.value,p)
  }
# Control for multiple comparison, Holm methodology. For cases where there is only 1 endpoint
this is obviously not needed.
  p.correct <- p.adjust(p.value,method="holm")
```

```
# Formatted output.
  name.data <- c("in-hive, 0","in-hive, 750","in-hive, 1500","in-hive, 3000","in-hive, 
6000","foragers, 0","foragers, 750","foragers, 1500","foragers, 3000","foragers, 6000")
  cat(paste(name.data[i],"\n",sep=""))
  names(p.correct) <- c("1h","2h","4h")
  print(dataabnbe[[i]])
  cat("\n")
  cat(paste(name.data[i],". Observed and expected binomial proportions.\n",sep=""))
  pab <- a[,-1]/a[,1]
  pab <- rbind(pab,pab[1,]+pab[2,]-pab[1,]*pab[2,])
  rownames(pab) <- c("TREAT.A","TREAT.B","TREAT.AB","Expected")
  print(pab)
  cat("\n")
  cat(paste(name.data[i],". Control of type I errors (Holm method) in binomial proportion 
test.\n",sep=""))
  print(p.correct)

  cat("-----------------------------------------------------------------\n")
}
```

ESM references

1. Dietemann V *et al.* 2013 Standard methods for varroa research. *J. Apic. Res.* **52**, 1–54. (doi:10.3896/IBRA.1.52.1.09)
2. Fries I *et al.* 2013 Standard methods for nosema research. *J. Apic. Res.* **52**, 1–28. (doi:10.3896/IBRA.1.52.1.14)
3. Delaplane KS, Steen J Van Der, Guzman-novoa E. 2013 Standard methods for estimating strength parameters of *Apis mellifera* colonies. *J. Apic. Res.* **52**, 1–12. (doi:10.3896/IBRA.1.52.1.03)
4. Medrzycki P *et al.* 2013 Standard methods for toxicology research in *Apis mellifera*. *J. Apic. Res.* **52**, 1–60. (doi:10.3896/IBRA.1.52.4.14)

5. OECD/OCDE. 1998 OECD Guideline 213 for the testing of chemicals: honeybees, acute oral toxicity test. (doi:10.1787/9789264070165-en)
6. Williams GR *et al.* 2013 Standard methods for maintaining adult *Apis mellifera* in cages under in vitro laboratory conditions. *J. Apic. Res.* **52**, 1–36. (doi:10.3896/IBRA.1.52.1.04)
7. Jeschke P, Nauen R, Gutbrod O, Beck ME, Matthiesen S, Haas M, Velten R. 2015 Flupyradifurone (Sivanto^{TM}) and its novel butenolide pharmacophore: Structural considerations. *Pestic. Biochem. Physiol.* **121**, 31–38. (doi:10.1016/j.pestbp.2014.10.011)
8. Nauen R *et al.* 2014 Flupyradifurone: a brief profile of a new butenolide insecticide. *Pest Manag. Sci.* **71**, n/a-n/a. (doi:10.1002/ps.3932)
9. US EPA. 2015 Notice of pesticide registration for flupyradifurone, EPA reg. no. 264-1143. Office of Pesticide Programs, Environmental Fate and Effects Division EFED, Environmental Risk Branch IV 1–6.
10. EFSA. 2016 Setting of new maximum residue levels for flupyradifurone in strawberries, blackberries and raspberries. *EFSA J.* **14**. (doi:10.2903/j.efsa.2016.4423)
11. Bayer CropScience AG. 2018 Sivanto worldwide. *www.sivanto.bayer.com/sivanto-worldwide.html*. See https://www.sivanto.bayer.com/sivanto-worldwide.html (accessed on 23 March 2018).
12. Campbell JW, Cabrera AR, Stanley-Stahr C, Ellis JD. 2016 An evaluation of the honey bee (Hymenoptera: Apidae) safety profile of a new systemic insecticide, flupyradifurone, under field conditions in Florida. *J. Econ. Entomol.* **96**, 875–878. (doi:10.1093/jee/tow186)
13. US EPA. 2014 Environmental fate and ecological risk assessment for foliar, soil drench, and seed treatment uses of the new insecticide flupyradifurone (BYI 02960).
14. Bayer CropScience AG. 2013 Flupyradifurone technical information.
15. US EPA. 2012 White paper in support of the proposed risk assessment process for bees.
16. EFSA. 2013 EFSA Guidance Document on the risk assessment of plant protection products on bees (*Apis mellifera*, *Bombus* spp. and solitary bees). *EFSA J.* **11**, 268. (doi:10.2903/j.efsa.2013.3295)
17. Crane E. 1975 *Honey. A comprehensive survey*. London: Heinemann.
18. Pierre J, Mesquida J, Marilleau R, Pham-Delègue MH, Renard M. 1999 Nectar secretion in winter oilseed rape, Brassica napus - Quantitative and qualitative variability among 71

genotypes. *Plant Breed.* **118**, 471–476. (doi:10.1046/j.1439-0523.1999.00421.x)

19. Knopper LD, Dan T, Reisig DD, Johnson JD, Bowers LM. 2016 Sugar Concentration in Nectar: A Quantitative Metric of Crop Attractiveness for Refined Pollinator Risk Assessments. *Pest Manag. Sci.* (doi:10.1002/ps.4321)
20. Syngenta. 2009 QuiltXcel - Broad spectrum fungicide for control of plant diseases.
21. US EPA. 2017 Propiconazole EC.
22. Knezevic S. 2017 *2017 Guide for weed, disease, and insect management in Nebraska*. The Board of Regents of the University of Nebraska–Lincoln.
23. Tosi S, Costa C, Vesco U, Quaglia G, Guido G. 2018 A 3-year survey of Italian honey bee-collected pollen reveals widespread contamination by agricultural pesticides. *Sci. Total Environ.* **615**, 208–218. (doi:10.1016/j.scitotenv.2017.09.226)
24. Milner AM, Boyd IL. 2017 Toward pesticidovigilance. *Science (80-.).* **357**, 1232–1234. (doi:10.1126/science.aan2683)
25. EFSA. 2012 Scientific Opinion on the science behind the development of a risk assessment of Plant Protection Products on bees (*Apis mellifera*, *Bombus* spp. and solitary bees). *EFSA J.* **10**, 1–275. (doi:10.2903/j.efsa.2012.2668)
26. EFSA. 2014 Towards an integrated environmental risk assessment of multiple stressors on bees : review of research projects in Europe, knowledge gaps and. **12**, 1–102. (doi:10.2903/j.efsa.2014.3594)
27. Russell RM, Robertson JL, Savin NE. 1977 POLO: A new computer program for Probit analysis. *Bull. Entomol. Soc. Am.* **23**, 209–213. (doi:10.1093/besa/23.3.209)
28. Cedergreen N. 2014 Quantifying synergy: A systematic review of mixture toxicity studies within environmental toxicology. *PLoS One* **9**. (doi:10.1371/journal.pone.0096580)
29. Backhaus T, Faust M. 2012 Predictive environmental risk assessment of chemical mixtures: a conceptual framework. *Environ. Sci. Technol.* (doi:10.1021/es2034125)
30. Belden JB, Gilliom RJ, Lydy MJ. 2007 How well can we predict the toxicity of pesticide mixtures to aquatic life? *Integr. Environ. Assess. Manag.* **3**, 364–372. (doi:10.1897/1551-3793(2007)3[364:HWCWPT]2.0.CO;2)
31. Lewis KA, Tzilivakis J, Warner DJ, Green A. 2016 An international database for pesticide risk assessments and management. *Hum. Ecol. Risk Assess.* **22**, 1050–1064. (doi:10.1080/10807039.2015.1133242)

32. Sgolastra F *et al.* 2017 Synergistic mortality between a neonicotinoid insecticide and an ergosterol-biosynthesis-inhibiting fungicide in three bee species. *Pest Manag. Sci.* **73**, 1236–1243. (doi:10.1002/ps.4449)

33. Tosi S, Nieh JC, Sgolastra F, Cabbri R, Medrzycki P. 2017 Neonicotinoid pesticides and nutritional stress synergistically reduce survival in honey bees. *Proc. R. Soc. B Biol. Sci.* **284**, 20171711. (doi:10.1098/rspb.2017.1711)

34. Folt C, Chen C. 1999 Synergism and antagonism among multiple stressors. *Limnol. Oceanogr.* **44**, 864–877. (doi:10.4319/lo.1999.44.3)

35. Deeks JJ, Higgins JP, Altman DG. 2011 Effect measures for dichotomous outcomes. In *Cochrane Handbook for Systematic Reviews of Interventions* (eds J Higgins, S Green),

36. Noordhuizen J, Frankena K, Thrusfield M, Graat E. 2001 *Application of quantitative methods in veterinary epidemiology*. Wageningen Pers.

37. R Core Team. 2016 R: a language and environment for statistical computing.

38. Robertson JL, Preisler HK, Russell RM. 2007 PoloPlus Probit and Logit analysis. *LeOra Softw.*

39. Sokal RR, Rohlf FJ. 1995 *Biometry: the principles and practice of statistics in biological research*. WH Freman and company: New York.

40. Wahl O, Ulm K. 1983 Influence of pollen feeding and physiological condition on pesticide sensitivity of the honey bee *Apis mellifera carnica*. *Oecologia* **59**, 106–128. (doi:10.1007/BF00388082)

41. Decourtye A, Lacassie E, Pham-Delegue M-H. 2003 Learning performances of honeybees (*Apis mellifera* L) are differentially affected by imidacloprid according to the season. *Pest Manag. Sci.* **59**, 269–278. (doi:10.1002/ps.631)

42. Meled M, Thrasyvoulou A, Belzunces LP. 1998 Seasonal variations in susceptibility of *Apis mellifera* to the synergistic action of prochloraz and deltamethrin. *Environ. Toxicol. Chem.* **17**, 2517–2520. (doi:10.1002/etc.5620171220)

43. Smirle MJ, Winston ML. 1987 Intercolony variation in pesticide detoxification by the honey bee (Hymenoptera: Apidae). *J. Econ. Entomol.* **80**, 5–8. (doi:10.1093/jee/80.1.5)

44. Baines D, Wilton E, Pawluk A, De Gorter M, Chomistek N. 2017 Neonicotinoids act like endocrine disrupting chemicals in newly-emerged bees and winter bees. *Sci. Rep.* **7**, 10979. (doi:10.1038/s41598-017-10489-6)
